# Stakeholder views of behavioral interventions for children and adolescents with obesity: Mega‐ethnography of qualitative syntheses

**DOI:** 10.1111/obr.13917

**Published:** 2025-03-19

**Authors:** Joanna Leaviss, Roos Verstraeten, Christopher Carroll, Andrew Booth, Munira Essat, Diana Castelblanco Cuevas

**Affiliations:** ^1^ Sheffield Centre for Health and Related Research University of Sheffield Sheffield UK; ^2^ European Commission–Joint Research Centre Ispra Italy

**Keywords:** behavior interventions, childhood obesity, mega‐ethnography, qualitative research

## Abstract

**Background:**

The prevalence of overweight and obesity among children and adolescents is rising and is a recognized global health problem. This overview of reviews explored the views of children, adolescents, and parents/caregivers regarding behavioral interventions for obesity management.

**Methods:**

Eleven electronic databases were searched to identify reviews of qualitative research regarding the views of children or adolescents with obesity, and their caregivers, concerning behavioral interventions for obesity. Synthesis was performed using a mega‐ethnography approach.

**Results:**

Eleven reviews were included. Factors associated with feasibility, acceptability, and equity were identified that influenced decisions to engage with these interventions. Children and adolescents with obesity can be encouraged to engage and participate in behavioral interventions if there is a positive environment, free from stigma; have the necessary resources needed to fully engage in the intervention; are taught holistic, practical skills that allow for long‐term lifestyle change, not just short‐term weight loss; and are provided with activities likely to be perceived as fun and enjoyable. Interventions are more acceptable to the child/adolescent when parents and families are able to engage with them.

**Conclusion:**

Practitioners can improve engagement with behavioral interventions for obesity management for children/adolescents if they are aware of specific motivating factors.

## 1 BACKGROUND

In 2022, an estimated 37 million children under the age of 5 were overweight or obese worldwide, and over 390 million children and adolescents aged 5–19 face the same issue.^1^, ^2^ The prevalence of overweight or obesity among children and adolescents has risen significantly over the past decades and is recognized as a major public health concern.^3^ Recent studies, using pooled or meta‐analysis, indicate that approximately one in five children or adolescents experience excess weight^4^ and estimate that in 2022, 65.1 million girls and 94.2 million boys aged 5–19 lived with obesity globally.^5^ Although overall prevalence rates in obesity continue to rise, this masks significant regional variations,^4^, ^6^ and they are forecast to continue increasing in both boys and girls over the next decade (World Obesity Atlas 2035).^7^ Previous research has demonstrated that excess weight in children and adolescents is linked to a complex interplay of inherent, behavioral, environmental, and sociocultural factors.^4^ The 2030 Agenda for Sustainable Development Goal (SDG) target 3.4 seeks to reduce premature mortality from noncommunicable diseases (NCDs) by one‐third by 2030, including obesity.^8^ The World Health Organization (WHO) recognized the need for normative guidance focusing on people‐centered integrated management and care of children and adolescents with obesity, supplementing existing prevention‐focused guidelines. This involves using a primary health care approach providing health and obesity management advice for children and their families. Behavioral interventions represent one possible approach in this context. To support these efforts, the WHO commissioned a series of reviews, including this one, which examines evidence concerning behavioral interventions for managing (not preventing) obesity in children and adolescents. The suite of reviews explored children and their parents/caregivers' views on a range of obesity management strategies, including surgical, physical activity, and dietary interventions. The current review focused on behavioral interventions. Behavioral interventions are interventions that are designed to effect behavior change in an individual, commonly used to apply to health behavior change. Although a number of interventions may fall under this definition, for the current review, we include behavioral interventions as defined below. We do not include diet and physical activity interventions as these are reported elsewhere.

For this review, “behavioral interventions” are defined as follows: (i) interventions that invoke behavioral models or theory; (ii) interventions based on psychotherapeutic models such as behavioral therapy, cognitive behavioral therapies, mindfulness, attitude and relationship techniques, psychotherapy, psychodynamic therapies, humanistic therapies, stimulus control, goal setting, and self‐monitoring; (iii) multimodal interventions—composite approaches to modifying behavior with more than one component. In the context of this review, “multimodal behavioral interventions” refer to therapeutic approaches that incorporate a combination of different methods, strategies, or modes of treatment. Instead of relying on a single intervention, they utilize a variety of (e.g., behavioral, dietary, physical activity, and educational) components, tailored to meet the diverse needs of individuals or populations.^9^ In the context of managing obesity, they commonly consist of dietary management, encouragement toward increased physical activity, and a reduction in sedentary behavior and are known as lifestyle interventions or weight management interventions; (iv) digital behavior change interventions—these may be delivered via mobile phones, smartphones, personal and desktop computers, wearable technology, or television.^10^ Recruitment to behavioral interventions is historically poor,^11^ with high attrition rates.^12^, ^13^ Although quantitative studies can assess the effectiveness of these interventions, they do not explore the possible reasons behind the apparent lack of acceptability, nor do they help us understand the barriers to behavior change in those for whom the intervention is not effective. Qualitative studies are valuable for understanding facilitators and barriers to initiating and adhering to behavioral interventions and to behavior change itself. This review is unique in exclusively exploring the perspectives of children and adolescents with obesity, as well as their parents or caregivers, regarding perceptions of behavioral interventions for obesity management. By understanding these perspectives, it may be possible to address factors influencing initiation and adherence, informing the development of future interventions.

### 1.1 Objective

This study aims to explore the views of children and parents or caregivers regarding behavioral interventions for children and adolescents with obesity.

## 2 METHODS

When preparing this review of reviews, we used EPOC's Protocol and Review Template for Qualitative Evidence Synthesis.^14^ The protocol is registered on PROSPERO, CRD42022313848.

### 2.1 Eligibility criteria

This was a mega‐ethnography; therefore, the only study design included was systematic reviews. The included reviews were assessed for quality using a formal tool (described below), and these assessments were used to reflect the richness and rigor of the findings. Therefore, although some reviews were judged to be at “high risk to rigor” in one or more areas (e.g., only searching one database), they were not excluded on that basis. We included qualitative reviews that included primary studies that had used recognized methods of qualitative data collection and qualitative data analysis. These may have been reported solely as qualitative reviews, or within mixed methods reviews of quantitative and qualitative studies. A minimum of one qualitative study was required for a review to be included. Reviews could be either published or unpublished in any language (Table 1).

**TABLE 1:** Problem definition (PerSPECTiF).

Perspective	Setting	Phenomenon of interest	Environment	Comparison	Time/timing	Findings
Children with obesity	Home	Behavioral interventions	High‐, middle‐, and low‐income countries	PROGRESS+ factors^b^, ^15^	Initiation	Themes
Adolescents^a^ with obesity	School				Continuation	Feasibility
Parents and caregivers	Clinical settings					Acceptability
						Equity

^a^ The term “Child” is defined by the WHO as a person aged 0–9 years old, an adolescent as a person 10–19 years old, and “youth” as one between the ages of 15 and 24 years old.^16^

^b^ Where reported, that is, any identified differences for PROGRESS+ factors.^15^

To be included in this review, reviews had to satisfy the topic of interest, defined here using the PerSPECTiF framework.^17^

Eligible behavioral interventions for obesity management met any of the broad definitions outlined in the background, namely, (i) interventions based on health behavior models or theories of behavioral change; (ii) interventions based on psychotherapeutic models; (iii) multimodal interventions; and (iv) digital interventions. Interventions could be delivered either individually or within a group setting and either via technologies (e.g., a mobile app or other computer‐mediated medium) or face‐to‐face. We excluded reviews that focussed on obesity prevention. The focus of this review was on qualitative data describing the views of children and adolescents and their parents or caregivers, in regard to behavioral interventions for obesity management, rather than the efficacy or effectiveness of any activities involved. The views of other adults involved in such activities, for example, clinicians, school teachers, or other staff, although acknowledged as important, are excluded from the scope of this review.

### 2.2 Search methods for identification of studies

A single search strategy was developed to populate all of the reviews in the suite (terms relating to childhood and adolescent obesity and qualitative reviews) with included reviews being mapped to the individual review topics during the sifting and mapping stage. An Information Specialist (AB), with extensive professional qualifications, developed the search strategies for 11 databases based on previous obesity reviews.^18^ These included African Journals Online (AJOL), ASSIA, CINAHL (Ovid), EMBASE (Ovid), EPISTEMONIKOS, Google Scholar, LILACS, MEDLINE (Ovid), PsycINFO (Ovid), Scopus, and Web of Science (WoS). No language limits were applied, but date coverage was restricted from January 2010 to ensure the most contemporaneous evidence (reviews published from 2010 onward would naturally include earlier relevant primary studies also). Searches were conducted on 01/23/2022. A methodological filter, used to populate the Cochrane Qualitative and Implementation Methods Group register of qualitative evidence syntheses, was applied to identify qualitative reviews. We conducted a gray literature search, examined previously identified WHO reviews,^18^ and reviewed the reference lists of included reviews and key references (i.e., relevant primary qualitative studies). We also undertook active pursuit of related studies, identified through shared authorship, citation networks, or related article features.^19^ See Appendix 1 in the Supporting Information for the elements of the search approach, the Ovid MEDLINE search strategy and all strategies used, and gray literature sources. An updated search was conducted in June 2024 using the Epistemonikos database, with a search strategy that is presented in Appendix 1 in the Supporting Information.

### 2.3 Selection of studies

After establishing acceptable agreement on the eligibility criteria through a test set of 100 references, the review team (CC, AB, ME, JL, ME, and DC) divided the remaining references between themselves. A second reviewer checked 10% of the Excludes for each reviewer, resolving discrepancies through team discussion. Full text was obtained for all potentially relevant papers identified by any reviewer. The lead reviewer and an independent additional reviewer then assessed these papers for final inclusion, resolving disagreements by discussion or, when required, by involving the senior author (AB).

### 2.4 Data extraction

We extracted data using a data extraction form designed for this qualitative evidence synthesis. The following data on review characteristics were extracted: author, year, aim of review, type of review, databases searched (names and number), inclusion criteria (age groups and regions/countries covered), number of included qualitative and mixed‐method studies (total number of included studies), appraisal tool used, type of synthesis conducted, and potential additional relevant references. The form also extracted synthesis data: relevant themes identified in the review, relevant themes identified by the authors of the primary studies (second‐order constructs), and any data from the primary studies supporting that theme (first‐order constructs). Some included reviews contained studies of multiple types of interventions, not of all which were behavioral interventions. Therefore only data from the relevant reviews were extracted, that is, those derived from the appropriate study design or intervention where a review may have included mixed quantitative or qualitative review, or a range of interventions including nonbehavioral interventions. As this was a suite of reviews covering a range of interventions, some reviews were included across more than one of our reviews. However, where this was the case, only data relating to behavioral interventions were extracted and are included here.

### 2.5 Assessing the methodological limitations of included studies

Methodological limitations of included qualitative evidence reviews were assessed using the *SBU assessment tool on Methodological Limitations of Qualitative Evidence Syntheses*.^20^ A copy of the tool is included in Appendix 5 in the Supporting Information. To our knowledge, this is currently the only tool available to assess the methodological limitations of qualitative evidence syntheses. One review author assessed the methodological limitations for each of the reviews using the SBU tool, and these assessments were cross‐checked by a second reviewer. Rare disagreements were resolved by discussion, without the involvement of a third review author. For evaluating the data richness in qualitative research, we applied a Qualitative Evidence Synthesis (QES) rating scale,^18^ which was modified from a scale originally developed for primary qualitative studies.^21^ This scale has a 3‐point score system that considers the quantity, depth, and relevance of qualitative data to the research question. A QES‐score of 3 indicates the highest data richness meaning it includes large quantities of qualitative studies (>20) or qualitative data (i.e., illustrative quotations from primary supporting studies), whereas a score of 2 or 1 indicates decreasing levels of data richness (2 indicates a substantive quantity of qualitative studies [10–20] or qualitative data and 1 indicates there are few qualitative studies [<10] or very little or no qualitative data). For rigor, >4 × high risk to rigor = High risk overall; <1 or no High risk to rigor; and <3 moderate risk = Low risk overall; and in the middle for moderate risk.

### 2.6 Data analysis and synthesis

Synthesis was performed using a mega‐ethnography approach.^22^ Mega‐ethnography requires the extraction of diverse findings from included reviews: first‐order constructs (any relevant participant verbatim comments from the original primary research studies); second‐order constructs (primary research authors' statements of findings); and third‐order constructs (themes emerging from the review authors' synthesis). Data on constructs were extracted independently by a single reviewer (JL) and checked by a second reviewer (RV), with discrepancies resolved through discussion (if necessary with a third reviewer [AB/CC]). Synthesis involved the interpretation of the first‐, second‐, and third‐order constructs by one reviewer (JL) to develop new, fourth‐order constructs, which were used to shape the review findings. Review findings were categorized according to qualitative aspects of the WHO evidence to decision‐making (EtD) framework,^23^ specifically the feasibility, acceptability, equity (FAE) domains (Table 2), which could facilitate the future implementation of interventions. Equity considerations were also addressed using the PROGRESS‐Plus components.^15^, ^24^ The GRADE EtD has up to nine items. However for this specific commissioned work, we were only considering a subset of the elements as described above and designated by WHO for the qualitative evidence, that is, those pertaining to feasibility, acceptability, and equity.

**TABLE 2:** Evidence to decision‐making (EtD) factors addressed by qualitative evidence.^23^

Feasibility	The likelihood that an intervention can be properly carried out or implemented in a given context
Acceptability	The extent to which an intervention is considered to be reasonable among those receiving, delivering, or affected by the intervention
Equity	Specific characteristics that are likely to be associated with disadvantage in relation to the review question being addressed. Equity considerations may be stated explicitly within the data or inferred from the description of context.

### 2.7 Review author reflexivity

As a review team, we were very aware of the need to consider our own biases when conducting this work. A full reflexivity statement is available in Appendix 2 in the Supporting Information.

## 3 RESULTS

### 3.1 Results of the search

**FIGURE 1:**
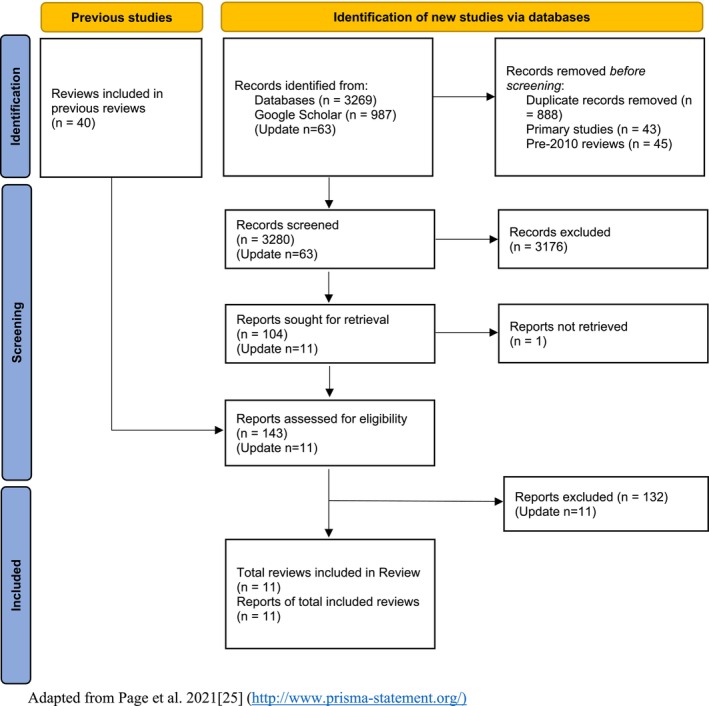
PRISMA flow diagram for this review.

Reviewers screened 3280 titles and abstracts, of which 143 were included for full‐paper screening. Eleven reviews were identified that satisfied the criteria for this review. The process is detailed in the PRISMA flow diagram (Figure 1). Appendix 3 (Supporting Information) lists studies excluded from the review at the full‐text stage and the main reason for exclusion.

### 3.2 Description of the included reviews

Details of the included reviews are presented in Tables 3 and 4. This review included 11 reviews published in English between 2013 and 2020. Seven reviews included both children and adolescents (age range: 0/2–18 years of age)^26^, ^27^, ^30^, ^31^, ^32^, ^33^, ^35^; three were mixed and included adults too^28^, ^34^, ^36^; and one focused exclusively on young people within the adolescent age group (aged 9–18 years).^29^ Where reviews contained studies of mixed child/adolescent/adult populations, only data pertaining to the child/adolescent studies were extracted.

**TABLE 3:** Reviews' characteristics (behavioral intervention synthesis).

First author (year of publication)	Review type	Age of children (years) (review inclusion criteria)	Intervention type	Regions^a^	Number of relevant qualitative studies (total studies in review)	Method of synthesis
Brigden (2020)^26^	Mixed methods synthesis	0–18	Digital behavior change interventions	Europe	3 (3)	N/R
Burchett (2018)^27^	Systematic review using QCA	0–11	Lifestyle weight management interventions	Europe	11 (11)	Thematic analysis
Grootens‐Wiegers (2020)^28^	Narrative synthesis	Mixed (child/adolescents with adults)	Group lifestyle interventions	NR	24 (24)	Overview of selected results
Jones (2019)^29^	QES	9–18	Lifestyle obesity treatments	North America, Europe, South East Asia, Western Pacific	24 (28)	Thematic synthesis
Kebbe (2017)^30^	Thematic synthesis	2–18	Healthy lifestyle behaviors	North America, Europe	3 (17)	Thematic synthesis
Kelleher (2017)^31^	Systematic review	2–18	Community‐based lifestyle programmes	North America, Europe, Western Pacific	8 (13)	Narrative synthesis
Lachal (2013)^32^	QES	0–18	Inpatient obesity treatment	Africa, North America, Central America, Eastern Mediterranean, Europe, Western Pacific	1 (45)	Meta‐synthesis, adapted from meta‐ethnography
Lang (2020)^33^	QES	2–18	Experiences of long‐term weight management	North America, Europe, South East Asia, Western Pacific	10 (16)	Thematic synthesis
Lyzwinski (2018)^34^	Meta‐ethnography	Mixed (child/adolescents with adults)	Mobile health interventions for weight loss	Americas, Western Pacific	6 (20)	N/R
Skelton (2014)^35^	Narrative synthesis	0–18	Pediatric obesity treatment	Europe	9 (18)	Narrative synthesis (includes scoping/mapping reviews)
Zarnowiecki (2020)^36^	Systematic review (unspecified)	Mixed (child/adolescents with adults)	Digital apps for improving child nutrition	Americas, Europe, Western Pacific	9 (35)	N/R

Abbreviations: NR, not reported; QCA, qualitative comparative analysis; QES, qualitative evidence synthesis.

^a^ WHO Regional Offices Classification: Africa, Americas, South East Asia, Europe, Eastern Mediterranean, and Western Pacific.

**TABLE 4:** Review methods (behavioral intervention synthesis).

First author (year of publication)	Search dates	Total number of databases listed	Main databases used	Additional databases	Supplementary search (i.e., nondatabase) methods	Quality assessment tool(s)	GRADE‐CERQual used
Brigden (2020)^26^	2014–January 2019	4	EMBASE, PsychINFO, MEDLINE, Cochrane Library	N/R	None reported	No (for qualitative data)	No
Burchett (2018)^27^	2012–December 9 and 10, 2015	11	CINAHL, MEDLINE, PubMed, PsycINFO, Web of Science	ASSIA (Proquest), Index to Theses (Proquest), British Education Index (EBSCO), ERIC (EBSCO), Health Management Information Consortium (OVID SP), Social Policy and Practice (OVID SP)	Existing NICE review	No	No
Grootens‐Wiegers (2020)^28^	July 2016	1	PubMed	None	Snowballing	No	No
Jones, (2019)^29^	Database inception to July 2018	6	MEDLINE, CINAHL, EMBASE, PsycINFO, ASSIA, Web of Knowledge/Science Citation Index/Social Science Citation Index	None	Relevant systematic reviews, key journals, and reference lists of included studies were manually screened. A specialist librarian was consulted to refine the search.	EPPI‐Centre Tool	No
Kebbe (2017)^30^	1980–June 2016	6	CINAHL, EMBASE, MEDLINE, PsycINFO, ProQuest Dissertations and Theses and Scopus	None	RLIS	MMAT	No
Kelleher, (2017)^31^	Database inception to 2015	4	MEDLINE, CINAHL, EMBASE, PsycINFO	None	RLIS	Other—Bowling's quality checklist	No
Lachal (2013)^32^	1990–2011	5	MEDLINE, CINAHL, EMBASE, PsycINFO, Scopus	None	RLIS	Other	No
Lang (2020)^33^	Database inception to January 2019; January 2019 to April 2020	6	MEDLINE in‐process and other nonindexed citations, EMBASE, CINAHL Plus, PsycINFO, Ovid Emcare, and Scopus	None	RLIS	CASP	No
Lyzwinski (2018)^34^	Searches: May 2016	4	PubMed (MEDLINE), CINAHL, Web of Science, Embase	None	N/R	Yes (CASP)	No
Skelton (2014)^35^	1990–2011	3	CINAHL, MEDLINE, PsycINFO	None	RLIS; studies by authors known in the field	No	No
Zarnowiecki (2020)^36^	Searches: 1946 to October 22, 2018. Studies restricted to January 1, 2013–October 2018	5	MEDLINE, EMCARE, PsychINFO, Scopus, ProQuest	None	RLIS	Yes (EPHPP)	No

Abbreviations: CASP, Critical Appraisal Skills Programme; EPHPP, Effective Public Healthcare Panacea Project; MMAT, Mixed Methods Assessment Tool; RLIS, Reference Lists of Included Studies.

Geographically, the majority of the evidence came from study populations in Europe (nine of the 11 reviews), followed by (North) America (seven out of 11 reviews) and the Western Pacific region (six out of 11 reviews). Only a limited number of reviews included study populations from Africa (one out of 11^32^) and South‐East Asia (two out of 11^29^, ^33^). One review did not specify the location of the included primary studies.^28^ Overall, the evidence came predominantly from high‐income countries. Across the analyzed reviews, a total of 230 primary qualitative studies were included and covered various interventions; 157 primary studies specifically related to behavioral interventions (Table 3).

The number of primary qualitative studies included in any review ranged from one^32^ to 24.^28^, ^29^ The included reviews searched between one^27^ and 11^28^ bibliographic databases (mean of five). The majority of reviews conducted a variant of thematic synthesis; four reviews did not include a quality assessment (Table 4).

Seven of the 11 reviews covered lifestyle weight management interventions, conducted either individually or in groups and at home or in community settings.^27^, ^28^, ^29^, ^30^, ^31^, ^32^, ^33^ One review explored inpatient obesity treatment,^35^ whereas the remaining three reviews focused on digital behavior change interventions, primarily delivered through mobile health applications (mHealth).^26^, ^34^, ^36^ Reviews of mHealth interventions predominantly explored perceptions of the usability and usefulness of these applications. No reviews exclusively focused on clinical interventions for children with obesity, and only two syntheses included interventions that were grounded in explicit health behavior models.^28^, ^33^

### 3.3 Methodological limitations of the reviews

Details of the quality and data richness assessments are presented in Table 5. The methodological assessment judged four of the reviews to have a minor risk to rigor.^26^, ^29^, ^30^, ^32^ For two reviews, the risk to rigor was moderate,^31^, ^34^ whereas five reviews were deemed to have a high risk to rigor.^27^, ^28^, ^33^, ^35^, ^36^ Two reviews were supported by rich data,^28^, ^29^ with one having a minor risk to rigor; two were supported by moderately rich data,^26^, ^33^ both assessed with a minor risk to rigor. The remaining seven reviews were considered to have meager data, with five of them at moderate or high risk to rigor.

**TABLE 5:** Quality assessment (risk to rigor evaluated using the SBU tool) and richness assessment for included reviews.

	Brigden (2020)^26^	Burchett (2018)^27^	Grootens‐Wiegers (2020)^28^	Jones (2019)^29^	Kebbe (2017)^30^	Kelleher (2017)^31^	Lachal (2013)^32^	Lang (2020)^33^	Lyzwinski (2018)^34^	Skelton (2014)^35^	Zarnowiecki (2020)^36^
1 Aim	L	L	L	L	L	L	L	M	L	M	L
2 Search approach	M	M	M	L	L	L	L	L	L	M	L
3 Inclusion criteria	L	L	H	L	L	L	L	L	L	L	L
4 Competence	M	M	M	M	L	M	L	L	M	M	H
5 Search strategy	L	M	M	L	L	L	L	M	M	L	L
6 Study screening	L	L	H	L	L	L	L	L	M	L	L
7 Appraisal tool	L	H	H	L	M	M	L	L	L	H	M
8 Appraisal process	L	H	H	L	L	M	M	L	M	H	L
9 Synthesis (method appropriateness)	L	L	M	L	L	L	L	H	L	H	M
10 Synthesis process	L	L	M	L	L	L	M	H	L	H	M
11 Synthesis output: clearly grounded in primary studies	L	H	M	L	L	L	L	H	L	L	H
12 Synthesis output: beyond a summary of primary studies	L	L	M	M	L	L	L	L	L	H	H
13 Confidence in finding (CERQual)	H	H	H	L	H	H	H	H	H	H	H
Overall verdict (concerns)	L	H	H	L	L	M	L	H	M	H	H
Data richness score	2	1	3	3	1	1	1	2	1	1	1

*Note*: Data richness: 3 = rich data; 2 = moderately rich data; 1 = meager data.

Abbreviations: H, high risk to rigor; M, moderate risk to rigor; L, low risk to rigor.

### 3.4 Review findings

A summary of the review findings (the fourth‐order constructs), and a list of the supporting reviews for each finding, grouped under the WHO EtD framework domains, is presented in Table 6. Full details of the findings of the synthesis: illustrative text extracts, the novel, fourth‐order constructs, their basis in the third‐order constructs of the included reviews, and the details of the reviews underpinning each finding, are presented in Appendix 4 (Tables S1–S3).

**TABLE 6:** Overall summary of qualitative findings (fourth‐order constructs).

Supporting reviews (number)	Findings
Feasibility of behavioral interventions (for full details see Table S1)	
Brigden (2020),^26^ Burchett (2018),^27^ Grootens‐Wiegers (2020),^28^ Jones (2019),^29^ Kebbe (2017),^30^ Kelleher (2017),^31^ Lang (2020)^33^ (*n* = 7)	*F1: Children and adolescents need parental support and a healthy home environment if they are to engage fully in behavioral interventions*. Facilitators: supporting the child, e.g., availability of healthy food Barriers: denial that the child needs to engage in weight loss intervention Family dynamics
Brigden (2020),^26^ Grootens‐Wiegers (2020),^28^ Jones (2019),^29^ Lang (2020)^33^ (*n* = 4)	*F2: Children and adolescents need effective support from health professionals and program staff when participating in behavioral interventions*. Attitude of the referrer Follow‐up support
Jones (2019),^29^ Kelleher (2017),^31^ Lang (2020)^33^ (*n* = 3)	*F3: A child or adolescent's relationships with their peers can influence their engagement with behavioral interventions for weight loss*.
Lang (2020)^33^ (*n* = 1)	*F4: The broader policy, educational, and community environment can affect the success of a behavioral intervention with children or adolescents*.
Kebbe (2017),^30^ Lang (2020)^33^ (*n* = 2)	*F5: The personal characteristics of children and adolescents can influence motivation to engage in behavioral interventions*. Biological or psychological factors Low self‐esteem Shame
Jones (2019),^29^ Kelleher (2017)^31^ (*n* = 2)	*F6: When overweight or obesity is stigmatized, children and adolescents with obesity are less motivated to engage with behavioral interventions*.
Acceptability of behavioral interventions (for full details, see Table S2)	
Jones (2019),^29^ Lyzwinski (2018),^34^ Zarnowiecki (2020)^36^ (*n* = 3)	*A1: Children and adolescents prefer behavioral interventions that are personalized and tailored to their needs*.
Brigden (2020),^26^ Grootens‐Wiegers (2020),^28^ Jones (2019),^29^ Kelleher (2017)^31^ (*n* = 4)	*A2: Children and adolescents prefer behavioral interventions that are fun and enjoyable*.
Brigden (2020),^26^ Lyzwinski (2018),^34^ Zarnowiecki (2020)^36^ (*n* = 3)	*A3: Children and adolescents prefer behavioral interventions delivered by mobile or digital technology when they see them as both useful and usable*.
Kelleher (2017),^31^ Lang (2020),^33^ Skelton (2014)^35^ (*n* = 3)	*A4: Children, adolescents, and their parents prefer behavioral interventions that are practical and solution‐based*.
Grootens‐Wiegers (2020),^28^ Zarnoweicki (2020),^36^ Kelleher (2017)^31^ (*n* = 3)	*A5: Behavioral interventions to children and adolescents should be delivered by a source that is perceived as trustworthy*.
Kelleher (2017)^31^ (*n* = 1)	*A6: Positive or negative attitudes or behaviors of staff delivering behavioral interventions influence whether children and adolescents feel motivated to continue to engage*.
Burchett (2018)^27^ (*n* = 1)	*A7: Provision of a safe space for children and adolescents to gain confidence and learn new skills is an important motivator to engage in behavioral interventions*.
Grootens‐Wiegers (2020),^28^ Kelleher (2017)^31^ (*n* = 2)	*A8: Parental concern about the consequences of their child's lifestyle can serve as either a motivator or a barrier to engagement in behavioral interventions*.
Grootens‐Wiegers (2020),^28^ Skelton (2014)^35^ (*n* = 2)	*A9: Children or adolescents value behavioral interventions that carry an expectation that they will achieve their desired outcomes*.
Burchett (2018),^27^ Jones (2019),^29^ Lang (2020)^33^ (*n* = 3)	*A10: It is important to children and adolescents that behavioral interventions stimulate a motivation and commitment to change*.
Kelleher (2017),^31^ Skelton (2014)^35^ (*n* = 2)	*A11: It is important to parents that behavioral interventions provide a holistic approach to weight loss for their children or adolescents*.
Kelleher (2017)^31^ (*n* = 1)	*A12: A family‐centered approach to behavioral interventions for children and adolescents is important to parents*.
Equity of behavioral interventions (for full details, see Table S3)	
Kelleher (2017),^31^ Lang (2020)^33^ (*n* = 2)	*E1: Access to appropriate facilities can influence the success of behavioral interventions for children and adolescents*.
Grootens‐Wiegers (2020),^28^ Kelleher (2017),^31^ Zarnoweicki (2020)^36^ (*n* = 3)	*E2: The cost of behavioral interventions for children and adolescents and their families can be a barrier to their uptake*. Characteristics of intervention Concerns over cost of technology
Jones (2019), Kelleher (2017)^31^ (*n* = 2)	*E3: It is important to acknowledge diversity when delivering behavioral interventions to children and adolescents*. Characteristics of children and their families Language difficulties Cultural differences
Grootens‐Wiegers (2020)^28^ (*n* = 1)	*E4: Personal circumstances may hinder the participation of children or adolescents in behavioral interventions*. Availability Lack of transport Lack of other resources

#### 3.4.1 Feasibility

##### Children and adolescents need parental support and a healthy home environment if they are to engage fully in behavioral interventions (F1; seven reviews including 39 primary studies)

Children and adolescents emphasize the crucial role of support at home, particularly from their parents. They rely on their parents to modify the home environment and provide resources to implement change.^33^ When parents acknowledge the role they play in supporting change, children and adolescents are motivated to initiate and adhere to behavioral interventions. Parental attitudes could either facilitate or act as a barrier to behavioral change; for example, parents' denial about adverse health implications of their child's overweight or underestimating the child's weight, can be a barrier in guiding them to an intervention.^28^ Family dynamics, including sibling report, play an important role in engagement with behavioral interventions, with potential conflicts and disagreements within families.^33^

##### Children and adolescents need effective support from health professionals and program staff when participating in behavioral interventions (F2; 4 reviews and 28 primary studies)

Children and adolescents with obesity, along with their parents, reported the need for professional support when participating in behavioral interventions. The quality of staff, including their experience, enthusiasm, and a positive staff–participant relationship were perceived as vital for sustained engagement; inadequate staff qualities could actually hinder efforts and lead to drop‐outs.^29^, ^33^ Constructive conversations about managing the child's weight or health were valued, but inconsistent advice or a lack of acknowledgment of participants' desire for support with weight loss were barriers to maintaining change.^33^

##### A child or adolescent's relationships with their peers can influence their engagement with behavioral interventions for weight loss (F3, 3 reviews including 32 primary studies)

A child or adolescent's relationship with their peers was found to influence their engagement with behavioral interventions. Peer support was valued,^29^ with participants appreciating it when peers acknowledged and respected their weight loss goals^33^; also, being surrounded by peers in similar situations reduced feelings of isolation.^27^ Group‐based programs provided additional social support by facilitating exchange of personal experiences, tips and tricks, and mutual accountability among participants (e.g.,^31^). Although parents were key to initial attendance, their children were the main drivers behind continued attendance.

##### The broader policy, educational, and community environment can affect the success of a behavioral intervention with children or adolescents (F4, 1 review, 10 primary studies)

Participants reported that community factors and public policy were important for positive engagement with behavioral interventions. In particular, access to sports facilities, parks, and nutritious food choices^33^ was seen as important, whereas “barriers to maintaining change included neighborhoods that did not support physical activity or limited access to healthy food choices.”^33^

##### The personal characteristics of children and adolescents can influence motivation to engage in behavioral interventions (F5; 2 reviews, 11 primary qualitative studies)

A child or adolescent's personal characteristics were reported to affect engagement. Biological or psychological factors were reported to influence emotional eating,^30^ making it more difficult for a child to follow a healthier diet plan, whereas low self‐confidence and low self‐esteem were often evident in children and adolescents participating in behavioral interventions,^33^ indicating their vulnerability.

##### The personal characteristics of children and adolescents can influence motivation to engage in behavioral interventions (F5; 2 reviews, 11 primary qualitative studies)

Children and adolescents are less motivated to engage in behavioral interventions if overweight or obesity is perceived to be stigmatized. Both children and their parents reported stigma around the child's weight as a barrier to initial attendance on weight management programs, as they did not want to be associated with such interventions.^31^, ^36^ Parental engagement was also influenced by external perceptions, with their decisions affected by societal perceptions, including those from close friends and family. Children also reported that feelings of failure, guilt, and shame served as a further barrier to their engagement in behavioral interventions.^29^

#### 3.4.2 Acceptability

##### Children and adolescents prefer behavioral interventions that are personalized and tailored to their needs (A1; 3 reviews, 16 primary studies)

Children and adolescents with obesity reported a dislike for vague, general information, much preferring if interventions were personalized and tailored to their own individual needs, including taking into account ethnicities, cultures, and age groups.^29^, ^34^, ^36^ Preference for personalization was reported for both digital health interventions^36^ and for lifestyle interventions for obesity.^29^ Unlike more general lifestyle interventions, digital health interventions/apps could be programmed to take into account individual preferences, for example, menu choices.

##### Children and adolescents prefer behavioral interventions that are fun and enjoyable (A2; 4 reviews, 28 primary studies)

Children reported that interventions that stimulate fun and enjoyment are valued and encourage active engagement.^29^, ^31^ For digital interventions, having a child‐centered design is important.^26^ For community‐based lifestyle interventions, “staff who made it fun” for children were found to enhance continued attendance.^31^

##### Children and adolescents prefer behavioral interventions delivered by mobile or digital technology when they see them as both useful and usable (A3; 3 reviews, 34 primary studies)

Both children and their parents reported key features of digital behavioral interventions that they considered important. Usability and usefulness were valued, with ease of use reported as both a facilitator and a barrier to engagement.^26^, ^36^ Additionally, features involving the whole family and were interactive were preferred by parents.^36^ Participants in one review reported how bland monotonous content, information, and delivery were barriers to engagement.^34^ Other important key features and functionality of digital interventions concerned the delivery of information: message type, timing and frequency, and personalisation.^34^

##### Children, adolescents, and their parents prefer behavioral interventions that are practical and solution‐based (A4; 3 reviews, 14 primary studies)

Children and adolescents and their parents reported that they preferred behavioral interventions that are practical and solution‐based. Learning new skills, especially problem‐solving skills, was reported to be important.^31^, ^33^ Attendance was reported to be enhanced by the inclusion of practical sessions for the chosen activities.^31^

##### Behavioral interventions to children and adolescents should be delivered by a source that is perceived as trustworthy (A5; 2 reviews, 33 primary studies)

Participants stressed the importance of trust when engaging in behavioral interventions, both in terms of the information being provided and the relationship with staff providing the activities.^28^, ^36^; good staff–participant relationships, as well as continuity of staff, were considered vital for continued attendance.^31^

##### Positive or negative attitudes or behaviors of staff delivering behavioral interventions influence whether children and adolescents feel motivated to continue to engage (A6; 1 review, 8 primary studies).

The positive or negative attitudes of staff delivering behavioral interventions were found to influence whether children and adolescents were motivated to engage: enthusiasm, knowledge, skills, and the ability to build relationships and communicate regularly were viewed as vital for continued engagement.^31^

##### Provision of a safe space for children and adolescents to gain confidence and learn new skills is an important motivator to engage in behavioral interventions (A7; 1 review, 5 primary studies)

The presence of a secure environment among peers was reported to be a pivotal motivator, determining the extent to which children and adolescents with obesity engage in behavioral interventions and enhancing their confidence and skills.^27^

##### Parental concern about the consequences of their child's lifestyle can serve as either a motivator or a barrier to engagement in behavioral interventions (A8; 2 reviews, 10 primary studies).

Parental concern for their child's health and well‐being, and the potential to identify medical issues as well as increasing self‐esteem were reported as a facilitator of initial engagement in two reviews.^28^, ^31^ However, in one of these reviews, parents also reported questioning the appropriateness or need for any such intervention for their child, rejecting the idea of any perceived weight‐related issues.^28^

##### Children or adolescents appreciate behavioral interventions that carry an expectation that they will achieve their desired outcomes (A9; 2 reviews, 7 primary studies)

Adherence to behavioral intervention was found to be highly dependent on whether expectations are met regarding, for example, weight loss or self‐confidence to make lifestyle changes.^28^, ^35^ Conversely, a perceived lack of weight loss may act as a barrier to adherence.

##### It is important to children and adolescents that behavioral interventions stimulate a motivation and commitment to change (A10; 3 reviews, 22 primary studies)

Participants value interventions that show you how to apply new skills and to change, not just what to change, and which provide long‐term support.^27^, ^29^ The challenges in initiating and maintaining change depend on agency, commitment, and achieving successes.^33^

##### It is important to parents that behavioral interventions provide a holistic approach to weight loss for their children or adolescents (A11; 2 reviews, 9 primary studies)

Parents are motivated to enroll their children in behavioral interventions if they are perceived to take a holistic approach to weight management, rather than focusing solely on diet with weight loss as the only goal.^31^, ^35^

##### A family‐centered approach to behavioral interventions for children and adolescents is important to parents and children (A12; 1 review, 4 primary studies)

Simultaneous delivery of interventions for both parents and children was highly valued and enhanced retention. This approach enabled active mutual support, shared experiences, and a sense of joy while collaborating, reducing the feeling of having to do everything by themselves.^31^ Additionally, including other family members (e.g., siblings) further increased the acceptability of the interventions.

#### 3.4.3 Equity

##### Access to appropriate facilities can influence the success of behavioral interventions for children and adolescents (E1; 2 reviews, 12 primary studies)

Children and adolescents need access to the appropriate, safe, and local facilities in order to engage in behavioral interventions.^31^ Conversely, limited access to physical activity resources or healthy food choices made initiating and maintaining change difficult.^33^ Challenges such as changing family circumstances, scheduling conflicts, and logistical issues, including distance and lack of public transport to program location, can hinder program attendance and lead to discontinuation.^31^ Parents often found it challenging to prioritize program time amid competing demands.

##### The cost of behavioral interventions for children and adolescents and their families can be a barrier to their uptake (E2; 3 reviews, 11 primary studies)

Costs associated with the purchase of relevant technology^36^ and arranging childcare to cover intervention attendance^28^ were influential to adherence. One review though concluded that involving the whole family in the intervention sometimes alleviated this added cost of childcare.^31^

##### It is important to acknowledge diversity when delivering behavioral interventions to children and adolescents (E3; 2 reviews, 12 primary studies)

Children and adolescents value interventions that are tailored to their personal characteristics, to their different ethnicities, cultures, and to specific age groups.^29^, ^31^

##### Personal circumstances may hinder the participation of children or adolescents in behavioral interventions (E4; 1 review, 1 primary study)

The personal and family circumstances of children and adolescents may affect their participation in behavioral interventions. These can include a lack of time, income, unavailability at specific meeting times, lack of transport, or lack of other resources.^28^

## 4 DISCUSSION

This overview of reviews draws insights from 11eleven reviews with a comprehensive global evidence base, encompassing over 150 primary qualitative studies. These insights are based mainly on evidence from high‐income countries.

Several factors were found to affect whether children and adolescents with obesity are prepared to consider, participate in, and continue with behavioral interventions delivered by health services.

Our qualitative evidence synthesis supports the importance of family involvement in the successful management of pediatric overweight or obesity, aligning with existing quantitative evidence, especially when siblings, grandparents, teachers, and friends' parents were involved.^37^ Furthermore, a qualitative evidence synthesis on weight stigma, although focused on adult populations, showed that stigma persists within interpersonal interactions between patients and healthcare providers; this stigma was found to be a barrier to equitable access to health services.^38^ Meanwhile, quantitative literature suggests that weight stigma is a key barrier to healthy behavior change in youth, as it may worsen obesity by inducing behavior such as binge eating, social isolation because of bullying, avoidance of health care services, and decreased physical activity.^39^, ^40^

Interventions were found to be most acceptable to children and adolescents when they were personalized, enjoyable, and practical; tailored to children and adolescents' own particular needs; and delivered through trustworthy sources. Children and adolescents and their parents or caregivers particularly preferred digital behavioral interventions and multicomponent approaches that are easy to use and perceived as useful. This finding is reflected in the wider literature, where high satisfaction and usability for pediatric telemedicine have also been found.^41^

In terms of equity, costs associated with accessing or attending interventions may disproportionately affect those with lower household budgets, whereas ease of access to appropriate facilities and resources can depend on where children and their families live. These findings are supported by a recent quantitative review, which revealed insufficient data on social and economic factors in pediatric obesity treatment administered through health services.^42^ The review underscores a failure to integrate contextual information from children with obesity and their families under treatment, limiting the development of effective and appealing interventions that encourage lasting change and adherence within the family's available resources. This is particularly concerning given the potential high costs and stigma experienced by patients in health care services, exacerbating disparities in access to and quality of obesity care in adult populations.^43^ Some of the findings for this review align with findings from other reviews done by the same research team. In particular, the perceived need for support from family, health professionals, and peers, along with the need for activities that are both personalized and more fun or interesting, has been found in both general and more intervention‐specific reviews of interventions.^18^, ^44^ However, other findings are quite distinct to the type of interventions covered here, in particular, first, the importance of mobile/digital technology and, second, the potential for greater acceptability of multimodal interventions beyond conventional diet and exercise directives.^45^ Childhood obesity can have a complex physiology, and long‐term behavior change can be challenging for any individual.^45^ The results of this review underscore the necessity for behavior change interventions for children and adolescents with obesity that invest in supportive environments at home, school, and the broader environment. Children and adolescents need a safe, positive space free from stigma in order to be motivated to initiate change. Awareness of certain potential health consequences of living with obesity can act as a motivator for both parents and children. This can also only be achieved where the family has adequate resources to adhere to and maintain behavioral change.

The current mega‐ethnography focuses on “obesity management.” Wider public health initiatives taking place further upstream to the population are classified as “obesity prevention.” Some of the views relating to obesity management may be shared with obesity prevention, and it may be useful in the future to explore similarities and differences between these two distinct types of intervention. We also did not explore the views of other adults, such as health workers, school teachers, or other education or social care workers, even though these are also important and their role was referenced by adolescents and caregivers in the included reviews.

## 5 LIMITATIONS OF THIS REVIEW

Although reviews included in this mega‐ethnography were required to be systematic, they varied in their quality, with some reviews conducting more comprehensive searches (i.e., in more databases) than others. Although eligibility criteria required one qualitative study to be included in source reviews for their inclusion in this overview of reviews, four of the reviews included full qualitative evidence syntheses.^29^, ^30^, ^32^, ^33^ These four reviews performed well for both methodological limitations (all minor concerns) and richness of data (two rich and two moderate) and made a major contribution to the findings. Although many findings from these four reviews were corroborated by reviews with more methodological limitations and less richness, this review would have been strengthened by the availability of a larger number of formally conducted qualitative evidence syntheses.

The GRADE EtD has up to nine items. However, for this specifically commissioned work, we were only considering a subset of those elements as designated by WHO (i.e., those pertaining to feasibility, acceptability, and equity).

Because of the size and scope of the review and the limited timescale, sifting, data extraction, and quality assessment were not conducted independently in pairs. This introduces a risk of bias and the authors acknowledge that the findings of this review are not fully reproducible. A series of methods was employed in order to mitigate this risk of bias. The six reviewers piloted each stage of the review and came together to ensure standardization. For the rigor and richness ratings, scores were compared between reviewers across common reviews in order to ensure external consistency. Nine reviews were included in more than one of the suite of reviews due to crossover of included studies (i.e., a review contained studies on, e.g., both physical activity and a behavioral intervention). Independent ratings, when compared, showed a very high level of consistency. There was only one difference in the overall assessment of rigor between all reviewers and five differences between reviewers for richness. These differences were resolved by consensus. However, we recognize that despite the measures taken, some assessments are largely subjective/interpretive, there is a risk of error, and bias will remain.

Findings were derived from 11 reviews with a global evidence base across 157 qualitative studies. Most of the evidence was collected in high‐income countries (e.g., USA)—therefore, further research from low‐ and middle‐income countries (LMICs), particularly those with a high share of deaths attributed to obesity, for example, Egypt, would add valuable perspectives. Although we aimed to explore PROGRESS+ factors within the analyses, there was only limited data reported in the included reviews on population characteristics such as gender, age, ethnicity, and socioeconomic status, and these factors were absent from the evidence and analysis in most existing reviews. Other factors such as disability were also not considered. The provision of such research data would facilitate a more granular analysis and a deeper understanding of factors that might apply to particular groups of young people.

## 6 CONCLUSIONS

This overview of reviews represents a global evidence base across more than 150 primary qualitative studies, primarily from high‐income countries. We found that some children and adolescents with obesity are motivated to engage with behavioral interventions if there is an expectation that they will lead to their desired outcome. Although the qualitative evidence within our review found that they also appreciate the delivery of such interventions using mobile or digital technology, there is currently only limited evidence for the effectiveness of such interventions, particularly with respect to impact on BMIz, weight, quality of life, depression, and anxiety. Motivation to initiate and adhere to these interventions is intricately linked to a range of factors, in particular, support from family, health professionals, and peers is important. Long‐term, holistic lifestyle change is considered more important than simple short‐term weight loss, and this is reflected in a preference for interventions that comprise practical problem‐solving and learning new skills. Multimodal behavioral interventions are also valued over more specific interventions, such as diet or exercise alone. A safe space for children free from guilt, shame, and stigma is vital.

## AUTHOR CONTRIBUTIONS

The protocol for this review was developed by the lead author (Joanna Leaviss) with all authors contributing to the final draft. Searches were undertaken by Andrew Booth, an experienced, qualified information professional using a standardized search strategy. Andrew Booth, Christopher Carroll, Joanna Leaviss, Munira Essat, and Diana Castelblanco Cuevas were involved in the screening of references and the selection of reviews. The included studies were divided among five of the authors (Andrew Booth, Christopher Carroll, Joanna Leaviss, Munira Essat, and Diana Castelblanco Cuevas) who extracted data and applied the SBU quality assessment tool to all their assigned reviews. Data extractions and quality assessments were checked by Andrew Booth. Synthesis was led by the lead author (Joanna Leaviss) with initial findings being discussed within the whole team (Andrew Booth, Christopher Carroll, Joanna Leaviss, Munira Essat, Diana Castelblanco Cuevas, and Roos Verstraeten). The lead author (Joanna Leaviss) drafted the first version of this report using a template adapted by Andrew Booth from the Cochrane EPOC QES review template. Andrew Booth was the principal investigator for the project and is the guarantor for this review.

## CONFLICT OF INTEREST STATEMENT

AB, CC, DC, JL, ME, and RV declared no financial conflicts of interest. AB, CC, JL, and ME conducted work under an exclusive contract with the World Health Organization. DC worked on the reviews under a Sheffield Centre for Health and Related Research (ScHARR), University of Sheffield internship. Nonfinancial issues, including personal, political, and academic factors, could have influenced the review authors' input when conducting this review. The review authors have discussed this further in the sections on reflexivity in the Methods and Results sections.

## Supporting information


**Data S1.** Supplementary Information.

## Data Availability

All data used in this review has been published in the included studies.
