# Supplementary material for: Stakeholder views of behavioral interventions for children and adolescents with obesity: Mega‐ethnography of qualitative syntheses
Source: Obes Rev. 2025 Mar 19;26(7):e13917. doi: 10.1111/obr.13917 (PMC12137028; doi:10.1111/obr.13917)
Supplement: Supplementary file 1 — Data S1. Supplementary Information. [file OBR-26-e13917-s001.pdf]

Title: Stakeholder views of behavioural interventions for children and adolescents with obesity: Mega-ethnography of qualitative syntheses

Authors:

*Joanna Leaviss, Sheffield Centre for Health and Related Research, University of Sheffield, Sheffield, UK

Roos Verstraeten, European Commission – Joint Research Centre, Ispra, Italy

Christopher Carroll, Sheffield Centre for Health and Related Research, University of Sheffield, Sheffield, UK

Andrew Booth, Sheffield Centre for Health and Related Research, University of Sheffield, Sheffield, UK

Munira Essat, Sheffield Centre for Health and Related Research, University of Sheffield, Sheffield, UK

Diana Castelblanco Cuevas, Sheffield Centre for Health and Related Research, University of Sheffield, Sheffield, UK

Running title: Views concerning behavioural interventions for children and adolescents with obesity

***Corresponding author**: Regent Court, Regent Street, Sheffield, S1 4DA j.leaviss@sheffield.ac.uk

Appendix 1: Search approach and strategy (Ovid MEDLINE)

STARLITE elements for documenting search approach

**Title: What matters to children, adolescents, parents and caregivers concerning behavioural interventions for children and adolescents with obesity?**	
**Sampling strategy**	Comprehensive: samples from international and regional databases
**Type of study**	Systematic reviews that include qualitative research studies
**Approaches**	Backwards citation chasing (reference checking) and Forwards citation chasing (Google Scholar citation searching using Publish or Perish)
**Range of years**	January 1, 2010 – December 2021
**Limits**	No language limits; Human only
**Inclusions and Exclusions**	Inclusion (Mega-aggregation): Reviews reporting perceptions of children, parents or caregivers concerning exercise and physical activity; desires, fears, experiences, coping strategies etc,: Reviews exploring initiation, contemplation or continuation of exercise and physical activity interventions or services
**Terms used**	See below for full strategy. Conceptualised as Obesity concepts AND Child/Adolescent/Parent terms AND Qualitative Review labels
**Electronic sources**	11 data sources: African Journals Online (AJOL); ASSIA; CINAHL (Ovid); EMBASE (Ovid); EPISTEMONIKOS; Google Scholar; LILACS; MEDLINE (Ovid); PsycINFO (Ovid); Scopus; Web of Science (WoS)

Search strategy (Ovid MEDLINE)

18 limit 17 to yr="2010 - 2021" 182

17 15 and 16 190

16 (Child or children or pediatr$ or paediatric$ or Child, Preschool or Adolescent$ or Adolescence or Youth or Youths or parent or parents or mother or mothers or father or fathers or relative or relatives or caregiver$ or Minors or minors$ or boy or boys or boyhood or girl$ or kid or kids or schoolchild$ or schoolchild or school child$ or juvenil$ or youth$ or teen$ or underage or pubescen$).mp. [mp=title, abstract, original title, name of substance word, subject heading word, floating sub-heading word, keyword heading word, organism supplementary concept word, protocol supplementary concept word, rare disease supplementary concept word, unique identifier, synonyms] 4745369

15 13 and 14 433

14 (obesity or obese or obesogenic).mp. [mp=title, abstract, original title, name of substance word, subject heading word, floating sub-heading word, keyword heading word, organism supplementary concept word, protocol supplementary concept word, rare disease supplementary concept word, unique identifier, synonyms] 363377

13 1 or 2 or 3 or 4 or 5 or 6 or 7 or 8 or 9 or 10 or 11 or 12 18685

12 (critical interpretive synthesis or (qualitative adj4 systematic$) or (qualitative adj4 review) or (qualitative adj4 synthes$)).mp. [mp=title, abstract, original title, name of substance word, subject heading word, floating sub-heading word, keyword heading word, organism supplementary concept word, protocol supplementary concept word, rare disease supplementary concept word, unique identifier, synonyms] 8756

11 (meta-study or metastudy or meta study or meta synthese or meta syntheses or meta synthesis or metasynthes$ or meta-synthes$ or meta synthesise or meta synthesised or meta synthesist or meta

synthesized or meta synthesizing or metasummar$ or meta-summar$ or meta summary or metanarrative$ or meta-narrative$ or meta narrative$).mp. [mp=title, abstract, original title, name of substance word, subject heading word, floating sub-heading word, keyword heading word, organism supplementary concept word, protocol supplementary concept word, rare disease supplementary concept word, unique identifier, synonyms] 1754

10 ((synthesis and ("qualitative literature" or "qualitative research")) or ("systematic review" and ("qualitative research" or "qualitative literature" or "qualitative studies"))).mp. [mp=title, abstract, original title, name of substance word, subject heading word, floating sub-heading word, keyword heading word, organism supplementary concept word, protocol supplementary concept word, rare disease supplementary concept word, unique identifier, synonyms] 4570

9 ((("quality assessment" or "critical appraisal" or checklist$) and ("mixed method" or "mixed methods" or "mixed studies" or "mixed study" or "mixed research")) or ("Mixed Methods Appraisal Tool" or MMAT)).mp. [mp=title, abstract, original title, name of substance word, subject heading word, floating sub-heading word, keyword heading word, organism supplementary concept word, protocol supplementary concept word, rare disease supplementary concept word, unique identifier, synonyms] 1041

8 (("literature search" or "literature searching" or "literature searches") and ("mixed method" or "mixed methods" or "mixed studies" or "mixed study" or "mixed research")).mp. [mp=title, abstract, original title, name of substance word, subject heading word, floating sub-heading word, keyword heading word, organism supplementary concept word, protocol supplementary concept word, rare disease supplementary concept word, unique identifier, synonyms] 213

7 ((("systematic review" or "systematic reviews") and ("mixed method" or "mixed methods" or "mixed studies" or "mixed study" or "mixed research")) or ((synthesis or syntheses) and ("mixed method" or "mixed methods" or "mixed studies" or "mixed study" or "mixed research"))).mp. [mp=title, abstract, original title, name of substance word, subject heading word, floating sub-heading word, keyword heading word, organism supplementary concept word, protocol supplementary concept word, rare disease supplementary concept word, unique identifier, synonyms] 2147

6 ((Noblit and Hare) or (CERQUAL or CONQUAL) or (JBI-QARI or QualSys)).mp. [mp=title, abstract, original title, name of substance word, subject heading word, floating sub-heading word, keyword heading word, organism supplementary concept word, protocol supplementary concept word, rare disease supplementary concept word, unique identifier, synonyms] 325

5 ((("quality assessment" or "critical appraisal" or checklist$) and ("qualitative literature" or "qualitative research" or "qualitative paper" or "qualitative papers" or "qualitative studies" or qualitative study or realist)) or (synthesis and ("qualitative literature" or "qualitative research")) or ("systematic review" and ("qualitative research" or "qualitative literature" or "qualitative studies"))).mp. [mp=title, abstract, original title, name of substance word, subject heading word, floating sub-heading word, keyword heading word, organism supplementary concept word, protocol supplementary concept word, rare disease supplementary concept word, unique identifier, synonyms] 5517

4 (("literature search" or "literature searching" or "literature searches") and ("qualitative literature" or "qualitative research" or "qualitative paper" or "qualitative papers" or "qualitative studies" or qualitative study or realist)).mp. [mp=title, abstract, original title, name of substance word, subject heading word, floating sub-heading word, keyword heading word, organism supplementary concept word, protocol supplementary concept word, rare disease supplementary concept word, unique identifier, synonyms] 676

3 (meta-triangulation or meta triangulation or meta triangulation or realist review or realist reviews or realist synthesis or realist syntheses or thematic synthesis or thematic syntheses or ((synthesis or syntheses) and Thematic analysis) or ((systematic review or systematic reviews) and

(Thematic analysis or framework synthesis or framework syntheses))).mp. [mp=title, abstract, original title, name of substance word, subject heading word, floating sub-heading word, keyword heading word, organism supplementary concept word, protocol supplementary concept word, rare disease supplementary concept word, unique identifier, synonyms] 2475

2 ((("integrative synthesis" or "integrative syntheses") and qualitative) or (("integrative review" or "integrative reviews") and qualitative) or ("interpretive synthesis" or "interpretive syntheses") or (Mega-ethnograph$ or megaethnograph$ or "mega ethnograph$" or (meta-ethnog$ or metaethnog$ or "meta ethnograph$") or ("meta interpretation" or "meta interpretive") or meta interpretation or meta interpretive or (Meta-method$ or "meta method$" or metamethod$) or "narrative synthesis" or "narrative syntheses")).mp. [mp=title, abstract, original title, name of substance word, subject heading word, floating sub-heading word, keyword heading word, organism supplementary concept word, protocol supplementary concept word, rare disease supplementary concept word, unique identifier, synonyms] 4656

1 ("Qualitative systematic review" or "qualitative systematic reviews" or "qualitative evidence synthesis" or "qualitative evidence syntheses" or "qualitative research synthesis" or "qualitative research syntheses" or "Qualitative synthesis" or "qualitative syntheses").mp. [mp=title, abstract, original title, name of substance word, subject heading word, floating sub-heading word, keyword heading word, organism supplementary concept word, protocol supplementary concept word, rare disease supplementary concept word, unique identifier, synonyms]

Search strategy - updated search (Epistemonikos, 2021 onwards).

(title:((title:((title:(obes* AND (child* OR adolescen* OR teen* OR youth* OR "young people" OR "young person*")) OR abstract:(obes* AND (child* OR adolescen* OR teen* OR youth* OR "young people" OR "young person*"))) AND qualitative) OR abstract:((title:(obes* AND (child* OR adolescen* OR teen* OR youth* OR "young people" OR "young person*")) OR abstract:(obes* AND (child* OR adolescen* OR teen* OR youth* OR "young people" OR "young person*"))) AND qualitative))) OR abstract:((title:((title:(obes* AND (child* OR adolescen* OR teen* OR youth* OR "young people" OR "young person*")) OR abstract:(obes* AND (child* OR adolescen* OR teen* OR youth* OR "young people" OR "young person*"))) AND qualitative) OR abstract:((title:(obes* AND (child* OR adolescen* OR teen* OR youth* OR "young people" OR "young person*")) OR abstract:(obes* AND (child* OR adolescen* OR teen* OR youth* OR "young people" OR "young person*"))) AND qualitative))))

Appendix 2: Reflexivity statement

During the screening at abstracts / titles and full text stages, the team constantly referred to each other team members to resolve conflicts. This review included positive, negative, and mixed perspectives in relation to the interventions of interest. As is standard practice within qualitative research, to minimise potential biases, reviewers (JL, RV) questioned each other's interpretation of the data and how it fitted with the interpreted findings. They also called upon other members of the review team (CC, AB, ME, DC) to verify that the findings were true reflections of the supporting data. The same was applied during the appraisal of confidence in the findings which was undertaken by JL and CC. Consultations with the contract editors of this review (JP, HP) represented a final measure to counter possible biases in the review findings.

Appendix 3: Studies excluded from the review when checking full text (identified from various sources) with main reason for exclusion

1. Abdin, S., Heath, G., & Welch, R. K. (2021). Health professionals' views and experiences of discussing weight with children and their families: A systematic review of qualitative research. Child: Care, Health and Development, 47(4), 562-574.	Excluded - only Health Professionals' views
2. Aboueid, S., Ahmed, R., Jasinska, M., Pouliot, C., Hermosura, B. J., Bourgeault, I., & Giroux, I. (2020). Weight Communication: How Do Health Professionals Communicate about Weight with Their Patients in Primary Care Settings?. Health Communication, 1-7.	Excluded - Not Adolescents
3. Aceves-Martins, M., López-Cruz, L., García-Botello, M., Gutierrez-Gómez, Y. Y., & Moreno-García, C. F. (2021). Interventions to Prevent Obesity in Mexican Children and Adolescents: Systematic Review. Prevention Science, 1-24.	Excluded - Not Treatment
4. Adamo, K. B., & Brett, K. E. (2014). Parental perceptions and childhood dietary quality. Maternal and child health journal, 18(4), 978-995.	Excluded - No Interventions
5. Adom T, De Villiers A, Puoane T, Kengne AP. School-Based Interventions Targeting Nutrition and Physical Activity, and Body Weight Status of African Children: A Systematic Review. Nutrients. 2019 Dec 30;12(1):95. doi: 10.3390/nu12010095. PMID: 31905832; PMCID: PMC7019429.	Excluded - Not Qualitative
6. Alman KL, Lister NB, Garnett SP, Gow ML, Aldwell K, Jebeile H. Dietetic management of obesity and severe obesity in children and adolescents: A scoping review of guidelines. Obesity Reviews [Internet]. 2020 Sep 7;22(1). Available from: http://dx.doi.org/10.1111/obr.13132	Excluded - Not Qualitative
7. Alsubhi, M., et al. (2020). What factors are associated with obesity-related health behaviours among child refugees following resettlement in developed countries? A systematic review and synthesis of qualitative and quantitative evidence. Obesity Reviews 21(11): e13058.	Excluded – No Interventions
8. Alulis, S., & Grabowski, D. (2017). Theoretical frameworks informing family-based child and adolescent obesity interventions: A qualitative meta-synthesis. Obesity Research & Clinical Practice, 11(6), 627-639.	Excluded – Focus on Theory not Intervention
9. Ames, H., et al. (2020). "Communication of children's weight status: what is effective and what are the children's and parents' experiences and preferences? A mixed methods systematic review." BMC Public Health 20(1): 574.	Excluded from Review 4 (no behavioural intervention data)
10. Arai, L., Panca, M., Morris, S., Curtis-Tyler, K., Lucas, P. J., & Roberts, H. M. (2015). Time, monetary and other costs of participation in family-based child weight management interventions: qualitative and systematic review evidence. PLoS One, 10(4), e0123782.	Excluded – Not Qualitative (Includes stand-alone primary qualitative study)

11. Archibald, D., Douglas, F., Hoddinott, P., Van Teijlingen, E., Stewart, F., Robertson, C., ... & Avenell, A. (2015). A qualitative evidence synthesis on the management of male obesity. BMJ open, 5(10), e008372.	Excluded - Not Adolescents
12. Bagnall, A. M., et al. (2019). Whole systems approaches to obesity and other complex public health challenges: a systematic review. BMC Public Health 19(1): 8	Excluded – No Interventions
13. Banna J, Bersamin A. Community involvement in design, implementation and evaluation of nutrition interventions to reduce chronic diseases in indigenous populations in the U.S.: a systematic review. Int J Equity Health. 2018 Aug 13;17(1):116. doi: 10.1186/s12939-018-0829-6. PMID: 30103753; PMCID: PMC6090789.	Excluded - Not Adolescents
14. Baranowski T, O'Connor T, Johnston C, Hughes S, Moreno J, Chen TA, Meltzer L, Baranowski J. School year versus summer differences in child weight gain: a narrative review. Childhood Obesity. 2014 Feb 1;10(1):18-24.	Excluded - Not Interventions
15. Bean MK, Caccavale LJ, Adams EL, Burnette CB, LaRose JG, Raynor HA, Wickham EP 3rd, Mazzeo SE. Parent Involvement in Adolescent Obesity Treatment: A Systematic Review. Pediatrics. 2020 Sep;146(3):e20193315. doi: 10.1542/peds.2019-3315.	Excluded RCTs only
16. Bennett L, Burns S. Implementing health-promoting schools to prevent obesity. Health Education. 2020 Jun 8;120(2):197-216.	Excluded – Health Promotion
17. Besson M, Gurviez P, Carins J. Using digital devices to help people lose weight: a systematic review. Journal of social marketing. 2020 Jul 30.	Excluded - Not Qualitative
18. Bianco, A., Jemni, M., Thomas, E., Patti, A., Paoli, A., Ramos Roque, J., Palma, A., Mammina, C. and Tabacchi, G., 2015. A systematic review to determine reliability and usefulness of the field-based test batteries for the assessment of physical fitness in adolescents—The ASSO Project. Int J Occup Med Environ Health, 28(3), pp.445-478.	Excluded - Not Qualitative
19. Biddle SJ, Petrolini I, Pearson N. Interventions designed to reduce sedentary behaviours in young people: a review of reviews. Br J Sports Med. 2014 Feb;48(3):182-6. doi: 10.1136/bjsports-2013-093078. Epub 2013 Dec 17. PMID: 24347578.	Excluded - Not Qualitative
20. Blower S, Swallow V, Maturana C, Stones S, Phillips R, Dimitri P, Marshman Z, Knapp P, Dean A, Higgins S, Kellar I, Curtis P, Mills N, Martin-Kerry J. Children and young people's concerns and needs relating to their use of health technology to self-manage long-term conditions: a scoping review. Arch Dis Child. 2020 Nov;105(11):1093-1104. doi: 10.1136/archdischild-2020-319103. Epub 2020 May 22. PMID: 32444448; PMCID: PMC7588410.	Excluded - Not Obesity
21. Boone, K, "Overcoming Improving Providers' Attitude in Addressing Obesity in Patients.", Georgia State University, 2020. https://scholarworks.gsu.edu/nursing_dnpprojects/25	Excluded, only Health Professionals' views

22. Bradbury D, Chisholm A, Watson PM, Bundy C, Bradbury N, Birtwistle S. Barriers and facilitators to health care professionals discussing child weight with parents: A meta-synthesis of qualitative studies. British Journal of Health Psychology. 2018 Sep;23(3):701-22.	Excluded, only Health Professionals' views
23. Braden, K. W. and C. R. Nigg (2016). Modifiable Determinants of Obesity in Native Hawaiian and Pacific Islander Youth. Hawai'i Journal of Medicine & Public Health : A Journal of Asia Pacific Medicine & Public Health 75(6): 162-171.	Excluded – No Interventions
24. Bristow C, Meurer C, Simmonds J, Snell T. Anti-obesity public health messages and risk factors for disordered eating: a systematic review. Health promotion international. 2020 Dec;35(6):1551-69.	Excluded - No Interventions
25. Bungay, H. and T. Vella-Burrows (2013). The effects of participating in creative activities on the health and well-being of children and young people: a rapid review of the literature. Perspectives in Public Health 133(1): 44-52.	Excluded – Not Included Intervention
26. Buru K, Emeto TI, Malau-Aduli AE, Malau-Aduli BS. The efficacy of school-based interventions in preventing adolescent obesity in Australia. In Healthcare 2020 Dec (Vol. 8, No. 4, p. 514). Multidisciplinary Digital Publishing Institute.	Excluded - Not Qualitative
27. Castronuovo, L., Guarnieri, L., Tiscornia, V., & Allemandi, L. (2021). Food Marketing, Eating Behaviors and Gender Among Children and Adolescents: A Scoping Review.	Excluded - No Interventions
28. Caughey, A. B., Sargeant, J. M., Møller, H., & Harper, S. L. (2021). Inuit country food and health during pregnancy and early childhood in the circumpolar north: a scoping review. International journal of environmental research and public health, 18(5), 2625.	Excluded - No Interventions
29. Chatham, R. E. and S. J. Mixer (2020). Cultural Influences on Childhood Obesity in Ethnic Minorities: A Qualitative Systematic Review. Journal of Transcultural Nursing 31(1): 87-99.	Excluded – No Interventions
30. Chatterjee A, Prinz A, Gerdes M, Martinez S. Digital Interventions on Healthy Lifestyle Management: Systematic Review. Journal of Medical Internet Research. 2021 Nov 17;23(11):e26931.	Excluded – Not Adolescents
31. Rosales Chavez JB, Garcia LM, Jehn M, Pereira MA, Bruening M. Relationship between different levels of the Mexican food environment and dietary intake: a qualitative systematic review. Public Health Nutr. 2020 Aug;23(11):1877-1888. doi: 10.1017/S1368980019004294. Epub 2020 Mar 27. PMID: 32216850.	Excluded – No Interventions
32. Chavez-Ugalde, Yanaina et al. "Conceptualizing the commercial determinants of dietary behaviors associated with obesity: A systematic review using principles from critical interpretative	Excluded – No Interventions

synthesis." Obesity science & practice vol. 7,4 473-486. 5 Apr. 2021, doi:10.1002/osp4.507	
33. Chriqui JF, Pickel M, Story M. Influence of school competitive food and beverage policies on obesity, consumption, and availability: a systematic review. JAMA pediatrics. 2014 Mar 1;168(3):279-86.	Excluded - Not Qualitative
34. Clarke, J., Fletcher, B., Lancashire, E., Pallan, M., & Adab, P. (2013). The views of stakeholders on the role of the primary school in preventing childhood obesity: a qualitative systematic review. Obesity Reviews, 14(12), 975-988.	Excluded – Health Promotion
35. Clarke, J. L. (2016). The role of the primary school in preventing childhood obesity (Doctoral dissertation, University of Birmingham).	Excluded - Not Qualitative
36. Confiac, N., et al. (2020). "Mexican American Parental Knowledge and Perceptions of Childhood Obesity: An Integrative Review." Hispanic Health Care International : The Official Journal of The National Association of Hispanic Nurses 18(2): 105-116.	Excluded – No Interventions
37. Coulman KD, MacKichan F, Blazeby JM, Owen-Smith A. Patient experiences of outcomes of bariatric surgery: a systematic review and qualitative synthesis. Obesity reviews. 2017 May;18(5):547-59.	Excluded - Not Adolescents
38. Cui Z, Seburg EM, Sherwood NE, Faith MS, Ward DS. Recruitment and retention in obesity prevention and treatment trials targeting minority or low-income children: a review of the clinical trials registration database. Trials. 2015 Dec;16(1):1-5.	Excluded - Not Qualitative
39. Curtis P, Thompson J, Fairbrother H. Migrant children within Europe: a systematic review of children's perspectives on their health experiences. Public Health. 2018 May;158:71-85. doi: 10.1016/j.puhe.2018.01.038. Epub 2018 Apr 5. PMID: 29627115.	Excluded - Not Obesity
40. Dankiw KA, Tsiros MD, Baldock KL, Kumar S. The impacts of unstructured nature play on health in early childhood development: A systematic review. Plos one. 2020 Feb 13;15(2):e0229006.	Excluded - Not Interventions
41. Dattilo AM, Carvalho RS, Feferbaum R, Forsyth S, Zhao A. Hidden realities of infant feeding: Systematic review of qualitative findings from parents. Behavioral Sciences. 2020 May;10(5):83.	Excluded - Not Interventions
42. Demers C, Brochu A, Higgins J, Gélinas I. Complex behavioral interventions targeting physical activity and dietary behaviors in pediatric oncology: A scoping review. Pediatric Blood & Cancer. 2021 Aug;68(8):e29090.	Excluded - Not Qualitative
43. Deyra M, Gay C, Gerbaud L, Berland P, Pizon F. Global health determinants perceived and expressed by children and adolescents	Excluded - Not Obesity

between 6 and 17 years: a systematic review of qualitative studies. Frontiers in Pediatrics. 2020 Apr 3;8:115.	
44. Dhaliwal, J., et al. (2014). "Attrition and the management of pediatric obesity: an integrative review." Childhood Obesity 10(6): 461-473.	Excluded - Focus not on Intervention but trial.
45. Diep CS, Foster MJ, McKyer EL, Goodson P, Guidry JJ, Liew J. What are Asian-American youth consuming? A systematic literature review. Journal of Immigrant and Minority Health. 2015 Apr;17(2):591-604.	Excluded - Not Interventions
46. Dixon BN, Ugwoaba UA, Brockmann AN, Ross KM. Associations between the built environment and dietary intake, physical activity, and obesity: A scoping review of reviews. Obesity Reviews. 2021 Apr;22(4):e13171.	Excluded - Not Interventions
47. Dixon, M. O. (2012). "Elementary school personnel's perceptions of and recommendations for managing child obesity: A naturalistic study." Dissertation Abstracts International Section A: Humanities and Social Sciences 73(3-A): 892.	Exclude – Not Child or Parental Perspectives
48. Enright, G., et al. (2020). "Effectiveness of Family-Based Behavior Change Interventions on Obesity-Related Behavior Change in Children: A Realist Synthesis." International Journal of Environmental Research & Public Health [Electronic Resource] 17(11): 08.	Excluded – no relevant data
49. Farnesi, B. C., et al. (2012). "Family-health professional relations in pediatric weight management: an integrative review." Pediatric Obesity 7(3): 175-186.	Excluded from Review 3 (no behavioural data)
50. Fraser, J., et al. (2011). Paternal Influences on Children's Weight Gain: A Systematic Review. Fathering: A Journal of Theory, Research, and Practice about Men as Fathers 9(3): 252-267.	Excluded – No Interventions
51. Harrison M, Brodribb W, Hepworth J. A qualitative systematic review of maternal infant feeding practices in transitioning from milk feeds to family foods. Maternal & child nutrition. 2017 Apr;13(2):e12360.	Excluded – No Interventions
52. Hesketh, K. R., Lakshman, R., & van Sluijs, E. M. (2017). Barriers and facilitators to young children's physical activity and sedentary behaviour: a systematic review and synthesis of qualitative literature. Obesity Reviews, 18(9), 987-1017.	Excluded – No Interventions
53. Hnatiuk JA, Brown HE, Downing KL, Hinkley T, Salmon J, Hesketh KD. Interventions to increase physical activity in children 0–5 years old: a systematic review, meta-analysis and realist synthesis. Obesity Reviews. 2019 Jan;20(1):75-87.	Excluded - Not Qualitative
54. Hoare JK, Jebeile H, Garnett SP, Lister NB. Novel dietary interventions for adolescents with obesity: A narrative review. Pediatric Obesity. 2021 May 5;16(9). Available from: http://dx.doi.org/10.1111/ijpo.12798	Excluded - Not Qualitative

55. Ibáñez, C. P. B., & Heredia, L. P. D. (2019). Características de las intervenciones realizadas con adolescentes en condición de sobrepeso y obesidad: una revisión integrativa. Revista Cuidarte, 10(3).	Excluded - Not Qualitative
56. Jebeile H, Lister NB, Baur LA, Garnett SP, Paxton SJ. Eating disorder risk in adolescents with obesity. Obesity Reviews. 2021 Jan 6;22(5). Available from: http://dx.doi.org/10.1111/obr.13173	Excluded - Not Qualitative
57. Kairey, L.1; Matvienko-Sikar, K.1; Kelly, C.2; McKinley, M. C.; O'Connor, E. M.4; Kearney, P. M.1; Woodside, J. V.3; Harrington, J. M.1- Supplement . T3P36 - Portion size in parents' eyes: a mixed methods systematic review of parental portioning practices for their children.	Excluded – No Interventions
58. Kanji, S., Wong, E., Akioyamen, L., Melamed, O., & Taylor, V. H. (2019). Exploring pre-surgery and post-surgery substance use disorder and alcohol use disorder in bariatric surgery: a qualitative scoping review. International Journal of Obesity, 43(9), 1659-1674.	Excluded – Not Adolescents
59. Keyworth C, Epton T, Goldthorpe J, Calam R, Armitage CJ. Delivering opportunistic behavior change interventions: A systematic review of systematic reviews. Prevention Science. 2020 Apr;21(3):319-31.	Excluded – Not Adolescents
60. Klingberg, S., Draper, C. E., Micklesfield, L. K., Benjamin-Neelon, S. E., & van Sluijs, E. M. (2019). Childhood obesity prevention in Africa: a systematic review of intervention effectiveness and implementation. International journal of environmental research and public health, 16(7), 1212.	Excluded – Not Adolescent Perspectives
61. Klingberg, S. (2020). Childhood obesity prevention in Soweto, South Africa (Doctoral dissertation, University of Cambridge).	Excluded – Not Adolescent Perspectives
62. Kobes A, Kretschmer T, Timmerman G, Schreuder P. Interventions aimed at preventing and reducing overweight/obesity among children and adolescents: a meta-synthesis. Obesity reviews. 2018 Aug;19(8):1065-79.	Excluded – Not Qualitative
63. König, L. M., Attig, C., Franke, T., & Renner, B. (2021). Barriers to and facilitators for using nutrition apps: systematic review and conceptual framework. JMIR mHealth and uHealth, 9(6), e20037.	Excluded – Not Adolescents
64. Kucharczuk AJ, Oliver TL, Dowdell EB. Social media's influence on adolescents′ food choices: A mixed studies systematic literature review. Appetite. 2022 Jan 1;168:105765.	Excluded – No Interventions
65. Lakshman, R., Griffin, S., Hardeman, W., Schiff, A., Kinmonth, A. L., & Ong, K. K. (2014). Using the Medical Research Council framework for the development and evaluation of complex interventions in a theory-based infant feeding intervention to prevent childhood obesity: the baby milk intervention and trial. *Journal of obesity*, 2014.	Excluded – No Interventions

66. Lampe, E. W., Abber, S. R., Forman, E. M., & Manasse, S. M. (2020). Guidelines for caregivers and healthcare professionals on speaking to children about overweight and obesity: A systematic review of the gray literature. Translational Behavioral Medicine, 10(5), 1144-1154.	Excluded – Not Qualitative
67. Lanigan J, Adegboye A, Northstone K, Salisbury C, Singhal A. Nutrition in preschool children and later risk of obesity: a systematic review and meta analysis. Journal of Pediatric Gastroenterology and Nutrition. 2016;62:691-2.	Excluded - Not Qualitative
68. Leandro, C. G., et al. (2019). "Barriers and Enablers That Influence Overweight/Obesity/Obesogenic Behavior in Adolescents From Lower-Middle Income Countries: A Systematic Review." Food & Nutrition Bulletin 40(4): 562-571.	Excluded – No Interventions
69. Leung, M. M., Cavalcanti, O. B., El Dada, A., Brown, M., Mateo, K. F., & Yeh, M. C. (2017). Treating obesity in Latino children: A systematic review of current interventions. International Journal of Child Health and Nutrition, 6(1), 1-15.	Excluded - Not Qualitative
70. Li, P. P., Mackey, G., Callender, C., Dave, J. M., Olvera, N., Alford, S., & Thompson, D. (2020). Culinary education programs for children in low-income households: A scoping review. Children, 7(5), 47.	Excluded – No Interventions
71. Littlewood, R., Canfell, O. J., & Walker, J. L. (2020). Interventions to prevent or treat childhood obesity in Māori & Pacific Islanders: a systematic review. BMC public health, 20(1), 1-14.	Excluded - Not Qualitative
72. Lofton, S., Julion, W. A., McNaughton, D. B., Bergren, M. D., & Keim, K. S. (2016). A systematic review of literature on culturally adapted obesity prevention interventions for African American youth. The Journal of School Nursing, 32(1), 32-46.	Excluded - Not Qualitative
73. Lu, W., McKyer, E. L. J., Lee, C., Goodson, P., Ory, M. G., & Wang, S. (2014). Perceived barriers to children's active commuting to school: a systematic review of empirical, methodological and theoretical evidence. International Journal of Behavioral Nutrition and Physical Activity, 11(1), 1-20.	Excluded - Not Qualitative
74. Ma, J., Lander, N., Eyre, E. L., Barnett, L. M., Essiet, I. A., & Duncan, M. J. (2021). It's not just what you do but the way you do it: a systematic review of process evaluation of interventions to improve gross motor competence. Sports Medicine, 51(12), 2547-2569.	Excluded – Not Obesity Outcomes
75. Mack I, Bayer C, Schaeffeler N, Reiband N, Broelz E, Zurstiege G, Fernandez-Aranda F, Gawrilow C, Zipfel S. Chances and limitations of video games in the fight against childhood obesity—A systematic review. European Eating Disorders Review. 2017 Jul;25(4):237-67.	Excluded - Not Qualitative

76. Zelenović M, Manić M, Stamenković A, Čaprić I, Božić D. Barriers to physical activity in adolescents: A systematic review. Turkish Journal of Kinesiology. 2021;7(1):22-30.	Excluded - Not Qualitative
77. Marks, R. (2015). Childhood obesity and parental health literacy. Advances in Obesity, Weight Management & Control, 3(3), 191-195.	Excluded – No Interventions
78. Marr, C., Reale, S., Breeze, P., & Caton, S. J. (2021). Grandparental dietary provision, feeding practices and feeding styles when caring for preschool-aged grandchildren: a systematic mixed methods review. Obesity Reviews, 22(4), e13157.	Excluded – No Interventions
79. Marshall, S., Taki, S., Laird, Y., Love, P., Wen, L. M., & Rissel, C. (2021). Cultural adaptations of obesity-related behavioral prevention interventions in early childhood: A systematic review. Obesity Reviews, e13402.	Excluded - Not Qualitative
80. Martins, J., Costa, J., Sarmento, H., Marques, A., Farias, C., Onofre, M., & Valeiro, M. G. (2021). Adolescents' perspectives on the barriers and facilitators of physical activity: an updated systematic review of qualitative studies. International Journal of Environmental Research and Public Health, 18(9), 4954.	Excluded – No Interventions
81. Matizanadzo, J. T., & Paudyal, P. (2021). The delivery of obesity interventions to children and adolescents with physical disabilities: a systematic review. Journal of Public Health.	Excluded – Not Adolescent/Parent Perspectives
82. Mawia, M. B. Tin Tin Su, Nik Daliana Nik Farid, Meram Azzani, Parents' Perception of Child Weight Status, Risk Factors and Health Concern of Childhood Obesity: A Systematic Review.(2020). Int. J. Life Sci. Pharma Res, 10(1), L15-32.	Excluded – No Interventions
83. McGill, B., Sweeting, J., Surkalim, D., Phongsavan, P., Thomas, M., & Bellew, W. (2020). New developments in the prevention of obesity among children and young people aged 0-18 Years: Rapid Evidence Review Update. New developments in the prevention of obesity among children and young people aged 0-18 years-Rapid evidence review update.	Excluded – Rapid Review of Reviews only
84. Messiah SE, Sacher PM, Yudkin J, Ofori A, Qureshi FG, Schneider B, Hoelscher DM, De la Cruz-Munoz N, Barlow SE. Application and effectiveness of eHealth strategies for metabolic and bariatric surgery patients: a systematic review. Digital health. 2020 Jan;6:2055207619898987.	Excluded – Not Adolescents
85. Militello LK, Kelly S, Melnyk BM, Smith L, Petosa R. A review of systematic reviews targeting the prevention and treatment of overweight and obesity in adolescent populations. Journal of Adolescent Health. 2018 Dec 1;63(6):675-87.	Excluded – Not Adolescent/Parent Perspectives
86. Moxthe, L. C., Sauls, R., Ruiz, M., Stern, M., Gonzalvo, J., & Gray, H. L. (2020). Effects of bariatric surgeries on male and female fertility: a systematic review. Journal of Reproduction & Infertility, 21(2), 71.	Excluded - Not Adolescents

87. Munt, A. E., Partridge, S. R., & Allman-Farinelli, M. (2017). The barriers and enablers of healthy eating among young adults: A missing piece of the obesity puzzle: A scoping review. Obesity reviews, 18(1), 1-17.	Excluded – Not Adolescents
88. Muzaffar, H., Metcalfe, J. J., & Fiese, B. (2018). Narrative review of culinary interventions with children in schools to promote healthy eating: directions for future research and practice. Current developments in nutrition, 2(6), nzy016.	Excluded – No Interventions
89. Ng CY, Thomas-Uribe M, Yang YA, Chu MC, Liu SD, Pulendran UP, Lin BJ, Lerner DS, King AC, Wang CJ. Theory-based health behavior interventions for pediatric chronic disease management: a systematic review. JAMA pediatrics. 2018 Dec 1;172(12):1177-86.	RCTs only
90. Nørnberg, T. R., Houlby, L., Skov, L. R., & Peréz-Cueto, F. J. A. (2016). Choice architecture interventions for increased vegetable intake and behaviour change in a school setting: a systematic review. Perspectives in public health, 136(3), 132-142.	Excluded - Not Obesity
91. Norris, E., Hamer, M., & Stamatakis, E. (2016). Active video games in schools and effects on physical activity and health: a systematic review. The Journal of Pediatrics, 172, 40-46.	Excluded - Not Qualitative
92. O'Connor, T., et al. (2018). "Engaging Latino Fathers in Children's Eating and Other Obesity-Related Behaviors: a Review." Current Nutrition Reports 7(2): 29-38.	Excluded – No Data
93. Ochieng, M. A. (2020). School nurses' nursing interventions in the prevention of childhood obesity. PhD Thesis	Excluded – Not Adolescent/Parent Perspectives
94. Ochoa, A. and J. M. Berge (2017). "Home Environmental Influences on Childhood Obesity in the Latino Population: A Decade Review of Literature." Journal of Immigrant and Minority Health 19(2): 430-447.	Excluded – No Interventions
95. Owusu, M, A Systematic Review Of The Interactions Between And Characteristics Associated With Obesity And Depressive Symptoms In The Pediatric Population" (2013). Yale Medicine Thesis Digital Library. 1827.<br>96. https://elischolar.library.yale.edu/ymtdl/1827<br>97. Dissertation Abstracts International: Section B: The Sciences and Engineering 74(12-B(E)).	Longitudinal cohort studies, cross-sectional studies of cohorts and intervention studies looking for treatment-associated changes
98. Ozodiegwu, I. D., et al. (2019). "A qualitative research synthesis of contextual factors contributing to female overweight and obesity over the life course in sub-Saharan Africa." PLoS ONE [Electronic Resource] 14(11): e0224612.	Excluded – No Interventions
99. Paes, V. M., et al. (2015). "Factors influencing obesogenic dietary intake in young children (0-6 years): Systematic review of qualitative evidence." BMJ Open 5(9).	Excluded – No Interventions

100. Park SH. Asian parents’ perception of child weight status: a systematic review. Journal of Child and Family Studies. 2017 Sep;26(9):2363-73.	Excluded – No Interventions
101. Patel, T., Umeh, K., Poole, H., Vaja, I., & Newson, L. (2021). Cultural identity conflict informs engagement with self-management behaviours for South Asian patients living with type-2 diabetes: A critical interpretative synthesis of qualitative research studies. International journal of environmental research and public health, 18(5), 2641.	Excluded – Not Adolescents
102. Pereira AI, Barros L. Parental cognitions and motivation to engage in psychological interventions: A systematic review. Child Psychiatry & Human Development. 2019 Jun;50(3):347-61.	Excluded – Not Adolescents
103. Pike KM, Dunne PE, Addai E. Expanding the boundaries: Reconfiguring the demographics of the “typical” eating disordered patient. Current psychiatry reports. 2013 Nov;15(11):1-8.	Excluded – Not Adolescent/Parent Perspectives
104. Pocock, M., et al. (2010). "Parental perceptions regarding healthy behaviours for preventing overweight and obesity in young children: a systematic review of qualitative studies." Obesity Reviews 11(5): 338-353.	Excluded – No Interventions
105. Pulgaron, E. R., et al. (2016). "Grandparent Involvement and Children's Health Outcomes: The Current State of the Literature." Families Systems & Health 34(3): 260-269.	Excluded – No Interventions
106. Pulimeno, Manuela et al. “Children's literature to promote students' global development and wellbeing.” Health promotion perspectives vol. 10,1 13-23. 28 Jan. 2020, doi:10.15171/hpp.2020.05	Excluded - No Interventions
107. Redsell SA, Slater V, Rose J, Olander EK, Matvienko-Sikar K. Barriers and enablers to caregivers' responsive feeding behaviour: A systematic review to inform childhood obesity prevention. Obesity Reviews. 2021 Jul;22(7):e13228.	Excluded – No Interventions
108. Rees, R., et al. (2011). "The views of young children in the UK about obesity, body size, shape and weight: a systematic review." BMC Public Health 11: 188.	Excluded – No Interventions
109. Rees, R. W., et al. (2014). "'It's on your conscience all the time': a systematic review of qualitative studies examining views on obesity among young people aged 12-18 years in the UK." BMJ Open 4(4): e004404.	Excluded – No Interventions
110. Regber, S. and H. Jormfeldt (2019). Foster homes for neglected children with severe obesity-Debated but rarely studied. Acta Paediatrica 108(11): 1955-1964.	Excluded – No Interventions
111. Reilly, J. J., Hughes, A. R., Gillespie, J., Malden, S., & Martin, A. (2019). Physical activity interventions in early life aimed at reducing later risk of obesity and related non-communicable diseases: A rapid review of systematic reviews. Obesity Reviews, 20, 61-73.	Excluded - Not Qualitative

112. Roberts, S. H., & Bailey, J. E. (2011). Incentives and barriers to lifestyle interventions for people with severe mental illness: a narrative synthesis of quantitative, qualitative and mixed methods studies. Journal of advanced nursing, 67(4), 690-708.	Excluded – Not Adolescents
113. Robertson C, Archibald D, Avenell A, Douglas F, Hoddinott P, van Teijlingen E, Boyers D, Stewart F, Boachie C, Fioratou E, Wilkins D. Systematic reviews of and integrated report on the quantitative, qualitative and economic evidence base for the management of obesity in men. Health Technology Assessment (Winchester, England). 2014 May;18(35):v.	Excluded – Not Adolescents
114. Ronto R, Rathi N, Worsley A, Sanders T, Lonsdale C, Wolfenden L. Enablers and barriers to implementation of and compliance with school-based healthy food and beverage policies: a systematic literature review and meta-synthesis. Public health nutrition. 2020 Oct;23(15):2840-55.	Excluded - No Interventions
115. Rose K, O'Malley C, Eskandari F, Lake AA, Brown L, Ells LJ. The impact of, and views on, school food intervention and policy in young people aged 11–18 years in Europe: a mixed methods systematic review. Obesity Reviews. 2021 May;22(5):e13186.	Excluded - No Interventions
116. Russell G, Laws R, Campbell K, Lynch J, Ball K, Denney-Wilson E. Parental influences on weight gain in infants and young children from disadvantaged families. Obesity Research & Clinical Practice. 2013(7):e122-3.	Excluded - No Interventions
117. Rylatt L, Cartwright T. Parental feeding behaviour and motivations regarding pre-school age children: A thematic synthesis of qualitative studies. Appetite. 2016 Apr 1;99:285-97.	Excluded - No Interventions
118. Sabate J, Wien M. Vegetarian diets and childhood obesity prevention. The American journal of clinical nutrition. 2010 May 1;91(5):1525S-9S.	Excluded - Not Qualitative
119. Sahota P, Wordley J, and Woodward J. Effective behavioural components in child and adolescent weight management programmes. *Obesity Reviews* 2011 Vol. 1) Pages 57-58	Excluded – Conference Abstract
120. Scott-Sheldon, L.A., Hedges, L.V., Cyr, C., Young-Hyman, D., Khan, L.K., Magnus, M., King, H., Arteaga, S., Cawley, J., Economos, C.D. and Haire-Joshu, D., 2020. Childhood Obesity Evidence Base Project: A systematic review and meta-analysis of a new taxonomy of intervention components to improve weight status in children 2–5 years of age, 2005–2019. Childhood Obesity, 16(S2), pp.S2-21.	Excluded - Not Qualitative
121. Singh A, Bassi S, Nazar GP, Saluja K, Park M, Kinra S, Arora M. Impact of school policies on non-communicable disease risk factors–a systematic review. BMC public health. 2017 Dec;17(1):1-9.	Excluded - No Interventions

122. Sosa, E. T. (2010). "Mexican American mothers' perceptions of childhood obesity and their role in prevention." Dissertation Abstracts International Section A: Humanities and Social Sciences 71(3-A).	Excluded – No Interventions
123. Sosa, E. T. (2012). "Mexican American mothers' perceptions of childhood obesity: a theory-guided systematic literature review." Health Educ Behav 39(4): 396-404.	Excluded – No Interventions
124. Stephen, A., Bermano, G., Bruce, D., & Kirkpatrick, P. (2013). Competencies and skills to enable effective care of severely obese patients undergoing bariatric surgery across a multi-disciplinary health care perspective: a systematic review protocol. JBI Evidence Synthesis, 11(8), 84-96.	Excluded - Protocol
125. Sutcliffe, K., Richardson, M., Rees, R., Melendez-Torres, G.J., Stansfield, C., Thomas, J., 2016. What are the Critical Features of Successful Tier 2 Weight Management Programmes?: A Systematic Review to Identify the Programme Characteristics, and Combinations of Characteristics, That are Associated with Successful Weight Loss. EPPI-Centre, UCL, London.	Excluded – Not Adolescents
126. Sutcliffe K., Melendez-Torres G.J., Burchett H.E.D., Richardson M., Rees R., Thomas J., The importance of service users' perspectives: a systematic review of qualitative evidence reveals overlooked critical features of weight management programmes, Health Expect., in preparation.	Excluded – Not Adolescents
127. Trübswasser, U., et al. (2020). "Factors influencing obesogenic behaviours of adolescent girls and women in low- and middle-income countries: A qualitative evidence synthesis." Obesity Reviews.	Excluded – No Interventions
128. van der Kleij RM, Coster N, Verbiest M, Van Assema P, Paulussen T, Reis R, Crone M. Implementation of intersectoral community approaches targeting childhood obesity: a systematic review. obesity reviews. 2015 Jun;16(6):454-72.	Excluded - No Interventions
129. Vanessa Augusta Souza, B., et al. (2017). "Nursing interventions with people with obesity in Primary Health Care: an integrative review." Revista da Escola de Enfermagem da USP 51: 1-10.	Excluded from Review 4 (no behavioural intervention)
130. Warr W, Aveyard P, Albury C, Nicholson B, Tudor K, Hobbs R, Roberts N, Ziebland S. A systematic review and thematic synthesis of qualitative studies exploring GPs' and nurses' perspectives on discussing weight with patients with overweight and obesity in primary care. Obes Rev. 2021 Apr;22(4):e13151. doi: 10.1111/obr.13151. Epub 2020 Dec 6. PMID: 33283435; PMCID: PMC7988601.	Excluded - Health professional perspectives
131. White B, Doyle J, Colville S, Nicholls D, Viner RM, Christie D. Systematic review of psychological and social outcomes of adolescents undergoing bariatric surgery, and predictors of success. Clinical obesity. 2015 Dec;5(6):312-24.	Excluded - Not Qualitative

132. Williamson C, Baker G, Mutrie N, Niven A, Kelly P. Get the message? A scoping review of physical activity messaging. International Journal of Behavioral Nutrition and Physical Activity. 2020 Dec;17(1):1-5.	Excluded – Not Adolescents
**Studies excluded when checking full text – updated search:**	
133. Ahmed, U., et al. (2023). "A Systematic Review Looking at the Current Best Practices as well as Primary Care Practitioner's Views on the Diagnosis and Treatment of Childhood Obesity." Cureus 15(1): e34346.	Excluded – outcomes are views of role of primary care in treatment
134. Arlinghaus, K. R., et al. (2022). "Outcomes From Healthy Eating and Physical Activity Recognition Programs in Early Child Care and Education: A Scoping Review." American journal of health promotion : AJHP: 8901171221116064.	Excluded - prevention
135. Baygi, F., et al. (2023). "The effect of psychological interventions targeting overweight and obesity in school-aged children: a systematic review and meta-analysis." BMC Public Health 23(1): 1478.	Excluded - quantitative
136. Bourke, M., et al. (2023). "Active video games and weight management in overweight children and adolescents-systematic review and meta-analysis." Journal of public health (Oxford, England) 45(4): 935-946.	Excluded - quantitative
137. Butscher, F., et al. (2024). "Influencing factors for the implementation of school-based interventions promoting obesity prevention behaviors in children with low socioeconomic status: a systematic review." Implementation science communications 5(1): 12.	Excluded - prevention
138. Chen, J., et al. (2024). "Reasons and promotion strategies of physical activity constraints in obese/overweight children and adolescents." Sports medicine and health science 6(1): 25-36.	Excluded – physical activity covered by alternate review
139. Ekambareshwar, M., et al. (2021). "Process evaluations of early childhood obesity prevention interventions delivered via telephone or text messages: a systematic review." Int J Behav Nutr Phys Act 18(1): 10.	Excluded - prevention
140. Lam, C., et al. (2022). "Internet of things-Enabled technologies as an intervention for childhood obesity: A systematic review." PLOS digital health 1(4): e0000024.	Excluded – outcomes not views
141. Shoesmith, A., et al. (2021). "Barriers and facilitators influencing the sustainment of health behaviour interventions in schools and childcare services: a systematic review." Implementation science : IS 16(1): 62.	Excluded - health behaviours are diet and physical activity, (covered in alternate review)
142. Skogen, I. B., et al. (2022). "Weight-Based Victimization and Physical Activity Among Adolescents With Overweight or Obesity: A Scoping Review of Quantitative and Qualitative Evidence." Frontiers in sports and active living 4: 732737.	Excluded – physical activity interventions (covered in alternate review)

Appendix 4: Supplementary tables S1-S3

Table S1: Summary of Qualitative findings: Factors relating to Feasibility

**First Author (year of publication)**	**Intervention**	**Number of qualitative studies**	**Third order constructs and primary study supporting statements/ illustrative quotations** (references cited in extracted text in this column are primary research studies being cited by included reviews)	**Fourth order constructs (Feasibility)**
Brigden (2020) (25)	Digital behaviour change interventions	2 (3)	Parental involvement:<br>*‘it does make me stop him and sit him down and make him eat the breakfast, rather than in the car’*<br>*‘made me more aware of how serious his asthma could get’*	**F1: Children and adolescents need parental support and a healthy home environment if they are to fully engage in behavioural interventions.**<br>- Facilitators: supporting the child e.g. availability of healthy food<br>- Barriers: denial that the child needs to engage in weight loss intervention<br>- Family dynamics
Burchett (2018) (31)	Lifestyle weight management interventions	3 (11)	Getting all the family ‘on-board’: Shared understanding and a healthy home environment	
Grootens-Wiegers (2020) (27)	Group lifestyle interventions	1 (24)	Intention to action – motivation:<br>"In order to prevent no show at the start of the programme, it is important that both the child and the parent are motivated to participate" (Grow et al., 2013)	
		1 (24)	Adherence – motivation:<br>"The parent’s commitment to the child’s health may be a strong motivator to overcome barriers during this stage" (Grow et al., 2013; Stewart et al., 2008)	
Jones (2019) (30)	Lifestyle obesity treatments	15 (28)	Support	
Kebbe (2017) (23)	Healthy lifestyle behaviours	1 (3)	Barriers: home environment – visibility in the home increased mindless eating	
		1 (3)	Enablers (Nutrition): Interpersonal:<br>Family, professional and social network<br>Text message delivery of favourite recipes	
Kelleher (2017) (24)	Community-based lifestyle programmes	3 (8)	Modifiable factors influencing initial attendance – Barriers: Parental denial:<br>Parental denial was another barrier to initial attendance (32,34,40). Parents sometimes relied on their own visual observation of their child rather than that of a health professional to justify rejecting a place on the associated weight management programme (34,40). These parents refused to accept their child was carrying excess weight with many referring to their child as ‘stocky’ or ‘broad’ (40), or believing they ‘would grow into it’ (34). Grow et al. (32) found that others compared their children to peers of similar build stating that they are	

First Author (year of publication)	Intervention	Number of qualitative studies	**Third order constructs and primary study supporting statements/ illustrative quotations** (references cited in extracted text in this column are primary research studies being cited by included reviews)	Fourth order constructs (Feasibility)
			‘normal, just like other children’ (34). This denial led to their perceived lack of need for such a programme and subsequently their refusal of the referral.	
Lang (2020) (22)	Experiences of long-term weight management	10 (10)	Interpersonal factor: Family dynamics:<br>The importance of their parents acknowledging the role they play in supporting change.<br>Providing encouragement and motivation.<br>Reliance on their family for additional support if they were “off track”.<br>Challenging family dynamics were often evident when parents, extended family, or siblings did not recognize that they play a role in supporting behaviour change.<br>Implementing lifestyle change could cause conflict, disagreements, and sibling rivalries within families.	
Lang (2020) (22)	Experiences of long-term weight management	10 (10)	Interpersonal factor: Family support:<br>Participants relied on their parents to make changes to the home environment and provide resources to implement change<br>Participants often relied on parents to role model healthy eating and lifestyle behaviours set rules, collaboratively change dietary behaviours, and increase activity<br>Families need to be equipped with the relevant skills to enable them to implement changes<br>Families often needed to adapt, adjust, and change their strategies if their current approach to behaviour change was not working<br>Some families did not make changes to the home environment that the young person required	
Brigden (2020) (25)	Digital behaviour change interventions	2 (3)	Connection with a health professional:<br>‘I think some kids will listen to their doctor better than their parents’<br>‘It should go back somehow to the pediatrician, some way, so that we’re almost held accountable during the visit.’<br>‘Communication…has been much more convenient’	**F2: Children and adolescents need effective support from health professionals and programme staff when participating in behavioural interventions.**<br>- Attitude of the referrer<br>- Follow-up support
Grootens-Wiegers (2020) (27)	Group lifestyle interventions	1 (24)	Initiation stage – referral:<br>Approach of the referrer: facilitation occurs when the approach is constructive, positive and solution-oriented, as opposed to being problem-oriented and judgemental (Mikhailovich & Morrison, 2007;	

First Author (year of publication)	Intervention	Number of qualitative studies	Third order constructs and primary study supporting statements/ illustrative quotations (references cited in extracted text in this column are primary research studies being cited by included reviews)	Fourth order constructs (Feasibility)
			Turner et al., 2012)<br>"(ii) attitude of the referrer: facilitation occurs if attitude is interested, sensitive, relational and patient-centred, but demotivating if distant and biomedically focused" (Edmunds, 2005; Edvardsson, Edvardsson, & Hornsten, 2009)<br>"(iii) language use of the referrer: facilitation occurs if language is positive and motivating, as opposed to blaming or stigmatizing" (Edvardsson et al., 2009; Puhl, Peterson, & Luedicke, 2011, 2013; Smith, Straker, McManus, & Fenner, 2014)<br>"(iv) focus of the conversation with the referrer: facilitation occurs if there is an awareness of the contextual complexity of overweight/obesity, but demotivating if an emphasis is placed on weight itself" (Edmunds, 2008; Mikhailovich & Morrison, 2007; Turner et al., 2012)	
Jones (2019) (30)	Lifestyle obesity treatments	15 (28)	Support	
Lang (2020) (22)	Experiences of long-term weight management	10 (10)	Interpersonal factor: Relationships with health care professionals:<br>Constructive conversations about managing their weight, health, and well-being with a healthcare professionals<br>Conversations with healthcare professionals about weight that were unhelpful, inconsistent advice, or a lack of acknowledgment of participants' desire for support with weight loss were barriers to maintenance of change	
		10 (10)	Institutional factor: Programs and services:<br>Some participants reported a lack of ongoing support or a sense of anxiety after completing a program or weight loss intervention	
Jones (2019) (30)	Lifestyle obesity treatments	17 (28)	Support	**F3: A child or adolescent's relationships with their peers can influence their engagement with behavioural interventions for weight loss (and A002)**
Kelleher (2017) (24)	Community-based lifestyle programmes	5 (8)	Modifiable factors influencing continued attendance – Facilitators:<br>Social interaction and support:<br>While parents were key to initial attendance, their children were the main drivers behind continued attendance.<br>Once enrolled in a programme, having fun (32,33,36) …and making new friends (32–34,38,40) motivated sustained engagement.	

First Author (year of publication)	Intervention	Number of qualitative studies	**Third order constructs and primary study supporting statements/ illustrative quotations** (references cited in extracted text in this column are primary research studies being cited by included reviews)	Fourth order constructs (Feasibility)
			Children particularly enjoyed the opportunity to play with children of a (i) similar age, (ii) weight status or (iii) activity level (32–34,38,40). Lucas et al. captured this point in the following quote where a participant expressed comfort in being surrounded by those of similar capability 'I found them fun because I was surrounded by different people who were in the situation that I was in, in terms of being overweight and finding exercise difficult.' (33).<br>Visram et al. (40) who evaluated an individual-based programme, as opposed to a group-based programme, reported that participating children stated they<br>were keen to meet other children in similar situations and recommended this as an area for improvement (40).<br>Parents returned to programmes primarily for the group support they received (32–34,38).<br>The shared experience often reduced feelings of 'isolation' (33)<br>…and many parents valued the 'social acceptance' of a group describing shared<br>problems which often resulted in the knowledge that they are not alone (33,38). While normalising the issue for many, these group-based programmes also offered further social support through the exchange of personal 'struggles and triumphs' (38), personal tips and tricks as well as holding each other accountable.<br>The parent-only session included in these programmes (32–34,38) allowed parents to discuss problems they may be experiencing in relation to their families positive lifestyle change with others on a similar journey that would not otherwise be possible in individual-based programmes.	
Lang (2020) (22)	Experiences of weight loss management	10 (10)	Interpersonal factor: Relationships with peers:<br>Acknowledgment and respect for a young person's weight loss goals from peers was considered supportive.<br>Collaboratively implementing dietary changes or increasing physical activity with peers was valued.<br>Peer pressure challenged their ability to maintain lifestyle change	
Lang (2020) (22)	Experiences of weight loss management	10 (10)	Institutional factor: Educational institutions/ employment:<br>Participants reported receiving support with behaviour change from within a school or university.	**F4: The broader policy, educational and community environment can affect the**

First Author (year of publication)	Intervention	Number of qualitative studies	Third order constructs and primary study supporting statements/ illustrative quotations (references cited in extracted text in this column are primary research studies being cited by included reviews)	Fourth order constructs (Feasibility)
			Employment could facilitate changes to routine and promote physical activity and adoption of healthy behaviors.<br>Sedentary employment, restrictive routines, and limited access to healthy food options in schools could impede maintenance of behavior change.	**success of a child or adolescent's behavioural intervention.**
		10 (10)	Community factors and public policy: The broader environment:<br>Access to sporting facilities, parks and nutritious food choices<br>Barriers to maintaining change included neighborhoods that did not support physical activity or limited access to healthy food choices<br>The broader social and cultural environment could influence participant's behavior	
Kebbe (2017) (23)	Healthy lifestyle behaviours	1 (3)	Barriers: biological and psychological factors – emotional eating	**F5: The personal characteristics of children and adolescents can influence motivation to engage in behavioural interventions.**<br>- Biological or psychological factors<br>- Low self-esteem<br>- Shame/stigma?
Lang (2020) (22)	Experiences of weight loss management	10 (10)	Vulnerable population: Vulnerability:<br>Participant's comments were often indicative of low self-confidence and self-esteem.<br>Anxieties regarding weight regain or reverting to previous lifestyle habits.<br>Weight-related stigma from peers, the broader community, and susceptibility to bullying.	
Kelleher (2017) (24)	Community-based lifestyle programmes	4 (8)	Modifiable factors influencing initial attendance – Barriers<br>Stigma:<br>The stigma surrounding the issue of excess weight and associated treatment programmes was reported as a significant barrier to initial attendance for both children and parents in four of the included studies (32–34,40).<br>Parents reported that children were reluctant to attend a programme for 'fat kids' either because they did not identify themselves as carrying excess weight or did not want others to identify them as being overweight (32).<br>Several children reported that they were hesitant to attend because they believed they were not 'fat' or because they disliked being identified by others as 'fat' (33).<br>The stigma surrounding the issue also appeared to influence whether or not parents engaged with a programme (33,34,40).	**F6: When overweight or obesity is stigmatised, children and adolescents with obesity are less motivated to engage in behavioural interventions.**

**First Author (year of publication)**	**Intervention**	**Number of qualitative studies**	**Third order constructs and primary study supporting statements/ illustrative quotations** (references cited in extracted text in this column are primary research studies being cited by included reviews)	**Fourth order constructs (Feasibility)**
			They appeared to be influenced by the perceptions held by close friends and family and were more likely to refuse referral if they expressed negative comments (34).<br>Parents were afraid of raising the subject of weight with their child out of fear of causing upset to them (32) or that involving them in such programmes would be harmful to their self-esteem (34,40).<br>Parental concerns about their child being labelled as overweight or obese and the negative impact on the child's self-esteem (40).	
Jones (2019) (30)	Lifestyle obesity treatments	11 (28)	Barriers to attending a weight management programme and being healthy: Prior fears of attending interventions; Obesity treatment bringing about feelings of failure, guilt and shame.	

Table S2: Summary of Qualitative findings: Factors relating to Acceptability

First Author (year of publication)	Intervention	Number of Qualitative studies	Third order constructs and primary study supporting statements/ illustrative quotations (references cited in extracted text in this column are primary research studies being cited by included reviews)	Fourth order constructs (Acceptability)
Jones (2019) (30)	Lifestyle obesity treatments	10 (28)	Tailored intervention (tailored to the individual, including different ethnicities, cultures and to the specific age group)	**A1: Children and adolescents prefer behavioural interventions that are personalised and tailored to their needs.**
Lyzwinski (2018) (28)	Mobile health interventions for weight loss	4 (6)	Personalisation	
Zarnoweicki (2020) (29)	Digital apps for improving child nutrition	2 (9)	Preferred content - personalisation	
Kelleher (2017) (24)	Community-based lifestyle programmes	5 (8)	Modifiable factors influencing continued attendance –<br>Facilitators: Social interaction and support:<br>Children enrolled to 'have fun' and 'make friends'<br>The opportunity to play games and exercise with others of a similar age<br>Parents enrolled with the expectation of meeting and gaining the support of other parents in the group	**A2: Children and adolescents prefer behavioural interventions that are fun and enjoyable.**
Brigden (2020) (25)	Digital behaviour change interventions	1 (3)	Child-centred design:<br>'It says please rate your fullness… I don't know how much [child] actually understands'<br>'irritating and annoying'<br>'I like eating from the plate'<br>'I like the electronic stuff'<br>'It's really confusing'<br>'I put my fingers in my ears' [regarding voice commands]<br>'boring' 'annoying'	
Jones (2019) (30)	Lifestyle obesity treatments	3 (28)	Active engagement (enjoyment and fun)	
		11 (28)	Physical activity vs diet: Enjoyment of sports and physical activity.	
		7 (28)	Technology: Adolescents enjoy using technology and do so with ease.	
Grootens-Wiegers (2020) (27)	Group lifestyle intervention	1 (24)	Adherence – satisfaction:<br>"Satisfaction with the intervention is based on: the focus of and activities in the programme" (Barlow & Ohlemeyer,	

			2006)<br>"...the relationship with the coaches and other participants" (Prioste, Fonseca, Sousa, Gaspar, & Francisco, 2015; Smith et al., 2014)<br>"A lack of trust or connection with coaches and participants or disliking activities or the group dynamics may be barriers to adherence" (Nobles et al., 2016)	
Brigden (2020) (25)	Digital behaviour change interventions	3 (3)	Technological affordances and barriers:<br>‘it’s just second nature really’<br>‘It’s not a huge disruption or anything’<br>‘You’ve got to faff around’<br>‘I think it’s hard, certain meals you have are harder to try and use it’<br>‘I think enthusiasm’s gone off. In the beginning it was a lot easier’<br>‘I don’t have to try to find the information. It comes to me.”<br>'I think the pro is that text is brief’<br>‘I didn’t start to text until the last year or two.”<br>‘They should know who has the [unlimited text messaging] plan…’<br>‘What if...I get to pick [the topics]. Don’t send me information about this…Every once in a while, you can throw some in, but I really want to focus on these.’<br>‘If you are getting these once a week and now its six weeks later and you haven’t really gotten any information that’s interesting to you, then I think I would text stop.’<br>‘Communication…has been much more convenient’<br>‘very easy…to keep track of everything’	**A3: Children and adolescents prefer behavioural interventions delivered by mobile or digital technology when they see them as both useful and usable.**
Lyzwinski (2018) (28)	Mobile health interventions for weight loss	1 (6)	Message tone	
		2 (6)	Message type	
		4 (6)	Message timing and frequency	
		1 (6)	Message content	
		2 (6)	Barriers – technology; cognitive/attentional (monotony of content, information and delivery)	
Zarnowiecki (2020) (29)	Digital apps for improving child nutrition	5 (9)	Preferred content:<br>Specific and relevant content preferred<br>Disliked general or vague information – wanted personalised and tailored<br>Desire for practical information to support behaviour change	

			Desire for trustworthy, evidence-based information<br>Desire for positive content, not a sole focus on obesity and weight management which elicited negative reactions such as shame and guilt	
		5 (9)	Preferred features and functionality:<br>Parents liked features that involved the whole family<br>Informative and practical<br>Preference for engaging and interactive features<br>Features that enable interaction with other users and health professionals	
		5 (9)	Functionality and delivery mode:<br>Library features with search function, customisable home page and personal user accounts<br>Information delivered by emails, text messages and social media<br>Mixed findings on use of push notifications, reminders and messaging	
		6 (9)	Usability, appeal and barriers:<br>Parents wanted digital tools to be self-explanatory, useful and easy to use<br>Cost was influential – tools should be low or no cost	
Kelleher (2017) (24)	Community-based lifestyle programmes	3 (8)	Modifiable factors influencing continued attendance – Facilitators: practical sessions:<br>Programmes which offered practical sessions further boosted continued attendance (32, 38,40).<br>These sessions, whereby parents tried new hands-on activities such as cooking demonstrations (32,38), healthy food shopping expeditions (38), visualising portion sizes (38), outdoor activity sessions (40), motivated families to continue attending.<br>Parents appreciated 'those kind of things, like the portion sizes... instead of maybe if the plate is this big, but actually show portion sizes to the parents so they can see it for themselves, see it being done' (38).<br>Results from Teevale et al.(38) suggest that parents were more interested in the practical aspect of the programme as opposed to the theory behind it. For example one mother reported that '...you don't want to hear theory when you're a	**A4: Children, adolescents and their parents value behavioural interventions that are practical and solution-based.**

			mum. You want to hear real-life experience and what's practical for us' (38).	
Lang (2020) (22)	Experiences of weight loss management	10 (10)	Intrapersonal factor: Managing the challenges of change:<br>Setting goals and planning ahead<br>Positive reinforcement to maintain change<br>Problem solving skills<br>Be mindful of food choices	
Skelton (2014) (26)	Paediatric obesity treatment	1 (2)	Parents thought patient-centred approaches with motivational interviewing were more favourable and child-friendly	
Grootens-Wiegers (2020) (27)	Group lifestyle intervention	1 (24)	Adherence – satisfaction	**A5: Behavioural interventions to children and adolescents should be delivered by a source that is perceived as trustworthy.**
Zarnoweicki (2020) (29)	Community-based lifestyle programmes	3 (9)	Preferred content	
Kelleher (2017) (24)	Community-based lifestyle programmes	3 (8)	Thrustworthy:<br>Conversely, a good staff–participant relationship was an important aspect of these programmes and viewed by some parents as vital for continued attendance (38,39).<br><br>Furthermore, Twiddy et al. (39) reported that the continuity of staff was important to the success of any programme as relationships can be built upon week after week (39).	
Kelleher (2017) (24)	Community-based lifestyle programmes	3 (8)	Modifiable factors influencing continued attendance – Facilitators: Programme staff:<br>Having staff who lack experience, enthusiasm or group management skills can hinder programme efforts and even result in some families dropping out of treatment.<br>Conversely, a good staff–participant relationship was an important aspect of these programmes and viewed by some parents as vital for continued attendance (38,39).<br>Staff 'who made it fun' for children and those with personal experience in either parenting or healthy weight management (33) enhanced continued attendance.<br>Furthermore, Twiddy et al. (39) reported that the continuity of staff was important to the success of any programme as relationships can be built upon week after week (39).	**A6: Positive or negative attitudes or behaviours of staff delivering behavioural interventions influences whether children and adolescents feel motivated to engage.**

			Regular communication between programme staff and families (38,40) where ‘study people would ring and remind’ parents further facilitated continued attendance (38).	
Burchett (2018) (31)	Lifestyle weight management interventions	5 (11)	Social support: a safe space with similar others in which to gain confidence and skills	**A7: Provision of a safe space for children and adolescents to gain confidence and learn new skills is an important motivator to engage in behavioural interventions.**
Kelleher (2017) 24)	Community-based lifestyle programmes	5 (8)	Modifiable factors influencing initial attendance – Facilitators: Parental concern for child’s well-being<br>Children just ‘went along’ without any particular reason<br>Parents were motivated to enrol largely because of concern for child’s health<br>….and their child’s psychological well-being<br>Child had been bullied<br>Opportunity to improve child’s self-esteem<br>Opportunity to improve child’s self-confidence<br>Mitigate adverse social experiences that child might be experiencing	**A8: Parental concern about the consequences of their child’s lifestyle can serve as either a motivator or a barrier to engagement in behavioural interventions.**
Grootens-Wiegers (2020) (27)	Group lifestyle interventions	5 (24)	(Health Belief Model) Initiation stage – motivation:<br>"Motivation of the child and parents can strongly influence the outcome of an attempt at guidance towards an intervention. Children and/or parents may be intrinsically concerned with the child’s weight" (Turner, Salisbury, & Shield, 2012)<br>"However, parents often underestimate the child’s weight, or the problematic nature of it, which can be a barrier in guiding them to an intervention" (Mikhailovich & Morrison, 2007).<br>"Other reasons for parents to be motivated to make lifestyle changes may be present, such as medical issues e.g., bad	

			teeth due to unhealthy diet" (Rietmeijer-Mentink, Paulis, van Middelkoop, Bindels, & van der Wouden, 2013)<br>Other reasons...."or social issues, such as a low self-esteem" (Stewart, Chapple, Hughes, Poustie, & Reilly, 2008)<br>Other reasons...."bullying or social exclusion" (Reece et al., 2015)	
Burchett (2018) (31)	Lifestyle weight management interventions	3 (11)	Learning how to change: Practical experiences that show you how to change, not only telling you what to change	**A9: Children and adolescents value behavioural interventions that stimulate a motivation and commitment to change.**
Jones (2019) (30)	Lifestyle obesity treatments	13 (28)	Maintenance	
Lang (2020) (22)	Experiences of long-term weight management	10 (10)	Intrapersonal factor: motivation versus ambivalence towards behaviour change:<br>Taking ownership of change.<br>Clarity regarding their desire for implementing and maintaining changes.<br>Commitment or determination to change.<br>Observing success from behaviour change.<br>Recognising others have been in similar situations is motivating.<br>Recognise need to change but did not initiate change.<br>Lose motivation if no tangible benefits.	
Grootens-Wiegers (2020) (27)	Group lifestyle intervention	2 (24)	Adherence - perceived benefits:<br>"...whether expectations are met" (Sallinen et al., 2013)<br>"Early treatment response may facilitate adherence" (Gunnarsdottir et al., 2011)<br>"Lack of weight loss may be a barrier to adherence" (Ward-Begnoche & Thompson, 2008)	**A10: It is important to children or adolescents that behavioural interventions will achieve their desired outcomes.**
		4 (24)	Intention to action – expectations:<br>"Expectations concerning the content of the intervention will be facilitating if potential participants and referrers are convinced that the activities in the programme are attractive and constructive" (Skelton & Beech, 2011)<br>".... and if the intervention is believed to lead to the desired outcome (e.g., weight loss or more self-confidence)" (Stewart et al., 2008)<br>"Expectations of one's behaviour play a role, and will be facilitating if participants expect to do well in the intervention" (Gunnarsdottir, Njardvik, Olafsdottir,	

			Craighead, & Bjarnason, 2011)<br>"....and feel confident that they will be able to make the lifestyle changes" (Gunnarsdottir et al., 2011)	
Skelton (2014) (26)	Paediatric obesity treatment	1 (2)	Parents were motivated to enroll based on perceived benefits to self-esteem and QOL rather than to improve weight outcomes	
Kelleher (2017) (24)	Community-based lifestyle programmes	7 (8)	Modifiable factors influencing initial attendance – Facilitators: Lifestyle focused approach<br>Parent's interest in programmes that focused on lifestyle.<br>Did not want their child to 'be put on a diet' – favoured a holistic approach.<br>Parents were interested in the informative part of the program.<br>Parents liked that it encompassed everything, the nutrition, the motivation and the exercise.<br>Parents cited the opportunity to learn new skills and enhance their knowledge on lifestyle related behaviours as further motivating factors.	**A11: It is important to parents that behavioural interventions provide a holistic approach to weight loss for their children or adolescents**
Skelton (2014) (26)	Paediatric obesity treatment	2 (unclear)	Parents were motivated to enroll based on perceived benefits to self-esteem and QOL rather than to improve weight outcomes	
Kelleher (2017) (24)	Community-based lifestyle programmes	4 (8)	Modifiable factors influencing continued attendance – Facilitators: Family-centred approach:<br>All of the included studies reported on family-based programmes where both parents and their child were invited to attend the sessions. This simultaneous delivery of the programme to parents and their children appeared to further enhance retention for a number of reasons (32,35,38).<br>Three of the included studies reported that both parents and children enjoyed the dedicated parent- child time that the programmes afforded (32,35,38)<br>either because they provided the opportunity to do exercise together or provided the mutual support they needed to keep attending. One parent expressed their appreciation of having 'something like that where it's just her and I doing something together, just the two of us, I mean I thought that was great' while another felt 'it was good opportunity for my child and me to do something together' (32).	**A12: A family-centred approach to behavioural interventions for children and adolescents is important to parents**

			Parents also placed value in a programme where both they and their child could attend together and therefore could actively participate and support each other (38).<br>Parents noted how receiving the same information made them ‘work together to help each other’ while others felt that ‘it would be hard’ to do the programme by themselves. One parent described ‘there was a time when my daughter would say, I don’t want to go, ’cause they’re telling me I can’t eat this and can’t eat that. And I go, No we’ll go, ’cause they’re telling me the same thing. When she saw it was difficult for me too and we started getting into a routine, she started wanting to go’ (38). Furthermore, inviting other family members to participate in these programmes boosted its acceptability (32,33,38,40).<br>Three of the included studies suggested inviting siblings to come along<br>as this sometimes alleviated the added cost of childcare (32,33,40).	

Table S3: Summary of Qualitative findings: Factors relating to Equity

**First Author (year of publication)**	**Intervention**	**Number of Qualitative studies**	**Third order constructs and primary study supporting statements/ illustrative quotations** (references cited in extracted text in this column are primary research studies being cited by included reviews)	**Fourth order constructs (Equity)**
Kelleher (2017) (24)	Community-based lifestyle programmes	2 (8)	Modifiable factors influencing initial attendance – Barriers and facilitators: Personal and programme logistics:<br>*Personal and programme logistics*<br>Finally, changing family circumstances such as moving school or relocating and scheduling conflicts were a challenge for many families (32,36).<br>Parents often found it hard to prioritise time for the programme when they had 'so many other things to do' in the evenings (34).<br>For others, programme logistics proved too difficult to overcome when deciding to enrol in a programme (32,34,36).<br>For example, in terms of location, both safety (34) and distance from home (32,36) were important factors influencing programme enrolment (32,34,35).<br>In addition to programme staff, logistical issues created significant barriers to continued attendance. Changing family circumstances including moving home, family illness or pregnancy (31–33,38)<br>Scheduling conflicts such as school holidays and after-school activities (32,33,35,38)<br>A lack of transport to programme location (32–35,38) were reported as reasons for families discontinuing care.<br>Lucas et al.(33) reported that transportation to the programme location was problematic when public transport was not available and driving not an option (33).	**E1: Access to appropriate facilities can influence the success of behavioural interventions for children and adolescents.**
Lang (2020) (22)	Experiences of weight loss maintenance	10 (10)	Community and public policy: the broader environment	

Grootens-Wiegers (2020) (27)	Group lifestyle intervention	5 (24)	Adherence – the means:<br>"The means to stay on the programme including: time, logistics and income" (Ligthart, Buitendijk, Koes, & van Middelkoop, 2016; Skelton et al., 2016; Smith et al., 2014)<br>"....support from the social environment that facilitates participation and lifestyle changes" (Denzer et al., 2004; Owen, Sharp, Shield, & Turner, 2009; Schalkwijk et al., 2015; Stewart et al., 2008)<br>" If participants need more support than the programme and the environment offer, this may lead to attrition" (Dhaliwal et al., 2014; Owen et al., 2009; Schalkwijk et al., 2015).	**E2: The cost of behavioural interventions for children and adolescents and their families can be a barrier to their uptake.**<br>Characteristics of intervention<br>- Concerns over cost of technology
Zarnowiecki (2020) (29)	User preferred content of digital apps targeting improvements in child's nutrition	2 (9)	Cost was influential – tools should be low or no cost	
Kelleher (2017) (24)	Community-based lifestyle programmes	4 (8)	Family-centred approach	
Jones (2019) (30)	Lifestyle obesity treatments	10 (28)	Tailored intervention	**E3: It is important to acknowledge diversity when delivering behavioural interventions to children and adolescents**<br>Characteristics of children and their families<br>- language difficulties<br>- cultural differences
Kelleher (2017) (24)	Community-based lifestyle programmes	2 (8)	Non-modifiable predictors of initial and continued attendance:<br>Language difficulties<br>Culturally inappropriate	

Grooten-Wiegers (2020) (27)	Group lifestyle intervention	1 (24)	Intention to action – means:<br>"The means of the potential participants may influence whether they are able to start an intervention. Barriers may include a lack of time, unavailability at specific meeting times, lack of transport or lack of other resources, such as not being able to find a sitter for other children in the family" (Smith et al., 2014)	**E4: Personal circumstances may hinder the participation of children or adolescents in behavioural interventions.**<br>- Availability<br>- Lack of transport<br>- Lack of other resources

Appendix 5. SBU tool

Tool to assess methodological limitations of qualitative evidence synthesis*

Author(s): ____________________ Year: ________

Title: ____________________ Reviewed by: ________

SUMMARY Minor concern ☐ Moderate concern ☐ High concern ☐

INTRODUCTION	YES	NO	NO INFO
1. AIM: Was the research question clearly stated?	☐	☐	☐
2. SEARCH APPROACH: Was the approach to searching for the literature appropriate for the research question?	☐	☐	☐
3. INCLUSION CRITERIA: Were the inclusion/exclusion criteria clearly described?	☐	☐	☐
4. COMPETENCE: Were there a sufficient number of researchers involved in the synthesis who had adequate competence?	☐	☐	☐
COMMENT:			

LITERATURE SEARCH AND SELECTION OF STUDIES	YES	NO	NO INFO
5. SEARCH STRATEGY: Was the search strategy sufficient to capture the relevant literature?	☐	☐	☐
6. STUDY SCREENING: Was the selection of relevant studies conducted independently by more than one reviewer and with consensus?	☐	☐	☐
COMMENT:			

APPRAISAL AND SYNTHESIS OF FINDINGS	YES	NO	NO INFO
7. APPRAISAL: Was risk of bias (or methodological quality) formally assessed using appropriate criteria?	☐	☐	☐
8. APPRAISAL PROCESS: Was the appraisal conducted independently by more than one reviewer and with consensus?	☐	☐	☐
9. SYNTHESIS: Was the synthesis method appropriate for the research question?	☐	☐	☐
10. SYNTHESIS: Was the synthesis conducted appropriately?	☐	☐	☐
11. SYNTHESIS OUTPUT: Were findings clearly grounded in the primary studies?	☐	☐	☐
COMMENT:			

SUMMARIZE THE CONCERNS IDENTIFIED DURING THE ASSESSMENT

Minor concern ☐ Moderate concern ☐ High concern ☐

Reason for concern ____________________

IF APPLICABLE...	YES	NO	NO INFO
A. SYNTHESIS OUTPUT: Did the synthesized result go beyond a summary of results from the included studies?	☐	☐	☐
B. CONFIDENCE IN FINDING: Was the confidence in the findings assessed with GRADE-CERQual in an appropriate way?	☐	☐	☐

* Tong A, Flemming K, McInnes E, Oliver S, Craig J. Enhancing transparency in reporting the synthesis of qualitative research: ENTREQ. BMC Med Res Methodol 2012;12:181.

SBU SWEDISH AGENCY FOR HEALTH TECHNOLOGY ASSESSMENT AND ASSESSMENT OF SOCIAL SERVICES

Reset form
